# Identification of an Activity Selector for the Nitroso‐Forming Activity in Bacterial Type‐III Copper Enzymes

**DOI:** 10.1002/anie.202501560

**Published:** 2025-05-24

**Authors:** Hoa Le Xuan, Felix Panis, Annette Rompel

**Affiliations:** ^1^ Fakultät für Chemie, Institut für Biophysikalische Chemie Universität Wien Josef‐Holaubek Platz 2 Vienna 1090 Austria; ^2^ Vienna Doctoral School in Chemistry (DoSChem) Universität Wien Währingerstraße 42 Vienna 1090 Austria

**Keywords:** Aminophenol, Biotechnology, C‐nitrosation, Enzyme engineering, Polyphenol oxidases

## Abstract

*O*‐aminophenol oxidases, a specialized subclass of type‐III copper proteins, play a crucial role in the biosynthesis of bioactive nitrosophenols, which display antiretroviral and blood cholesterol‐lowering activity. Another related subclass of type‐III copper proteins, tyrosinases, closely resemble *o*‐aminophenol oxidases structurally and enzymatically but lack their unique ability to oxidize *o*‐aminophenols into nitrosophenols. To unpuzzle the catalytic disparities of both subclasses, highly conserved amino acid residues in the vicinity of the catalytic center were identified. Notably, the Asn43 residue in the *o*‐aminophenol oxidase from *Streptomyces griseus* (*Sg*GriF) plays a pivotal role in its nitroso‐forming activity. Mutating the Asn43 residue in *Sg*GriF to isoleucine, which is present at the homologous position Ile42 in the tyrosinase from *Streptomyces* sp. ZL‐24 (*Sz*TYR), resulted in the loss of the nitroso‐forming activity in *Sg*GriF. Conversely, exchanging Ile42 in *Sz*TYR to asparagine generates nitroso‐forming activity in *Sz*TYR. The results presented herein demonstrate the feasibility of converting an *o*‐aminophenol oxidase into a tyrosinase and vice versa through a single amino acid mutation, underscoring the potential of these findings for future applications in medicinal and material sciences.

## Graphical abstract

**Small but crucial difference**: Type‐III copper enzymes, like tyrosinase (TYR) and *o*‐aminophenol oxidase (AO), catalyze the oxidation of *o*‐aminophenols (AP) to quinone imines. Unlike TYR, AO can further oxidize AP to nitrosophenols. This nitroso‐forming activity is controlled by a key amino acid near the Cu(A) ion. Mutating Asn to Ile in the AO *Sg*GriF converts it to a TYR, while the homologous Ile to Asn mutation in *Sz*TYR switches it from a TYR to an AO.

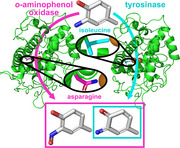

Type‐III copper proteins, which include tyrosinases (TYRs, EC 1.14.18.1) and *o*‐aminophenol oxidases (AOs, EC 1.10.3.4) feature a dicopper center within their catalytic site that supposedly binds dioxygen in a side‐on (*μ*‐*η* ^2^∶*η* ^2^) fashion.^[^ ^1^, ^2^, ^3^ ^]^ Type‐III copper enzymes exist in all domains of life, from archaea and bacteria to fungi, plants, and animals.^[^ ^4^, ^5^, ^6^, ^7^ ^]^ TYRs hydroxylate monophenols to the corresponding *o*‐diphenols (monophenolase activity, Figure S1) and oxidize *o*‐diphenols to *o*‐quinones (diphenolase activity, Figure S1).^[^ ^1^, ^8^ ^]^ Moreover, TYRs also accept *ο*‐aminophenol substrates (**1**, Figure 1: **reaction scheme A**), which can be oxidized in a two‐electron oxidation to *o*‐quinone imines (**2**, quinone imine‐forming activity, Figure 1: **reaction scheme A**).^[^ ^9^, ^10^ ^]^ Those *o*‐quinone imines (**2**, Figure 1: **reaction scheme A**) dimerize non‐enzymatically to phenoxazinones (**3**, Figure 1: **reaction scheme A**).^[^ ^9^, ^10^ ^]^ Similar to TYRs, AOs exhibit monophenolase (TYR, Figure S1) and diphenolase activity (TYR, CO, Figure S1) as well as quinone imine‐forming activity (Figure 1: **reaction scheme A**), but in contrast to TYRs, AOs are capable of oxidizing *o*‐aminophenols (**1**) into nitrosophenols (**7**, nitroso‐forming activity, Figure 1: **reaction scheme B**).^[^ ^3^, ^11^ ^]^ Nitrosophenols have been identified as biologically active, exhibiting antiatherosclerotic activity by inhibiting cholesteryl ester transfer protein.^[^ ^12^, ^13^, ^14^, ^15^ ^]^ Additionally, C‐nitroso compounds hold potential applications as precursors or catalysts in organic synthesis and as additives for the functionalization of natural products.^[^ ^16^, ^17^, ^18^ ^]^ These compounds are also utilized in the synthesis of branched nitrogen‐containing polymers, and polymeric nitroso compounds can be formed under mild oxidation conditions.^[^ ^19^, ^20^ ^]^

**Figure 1:**
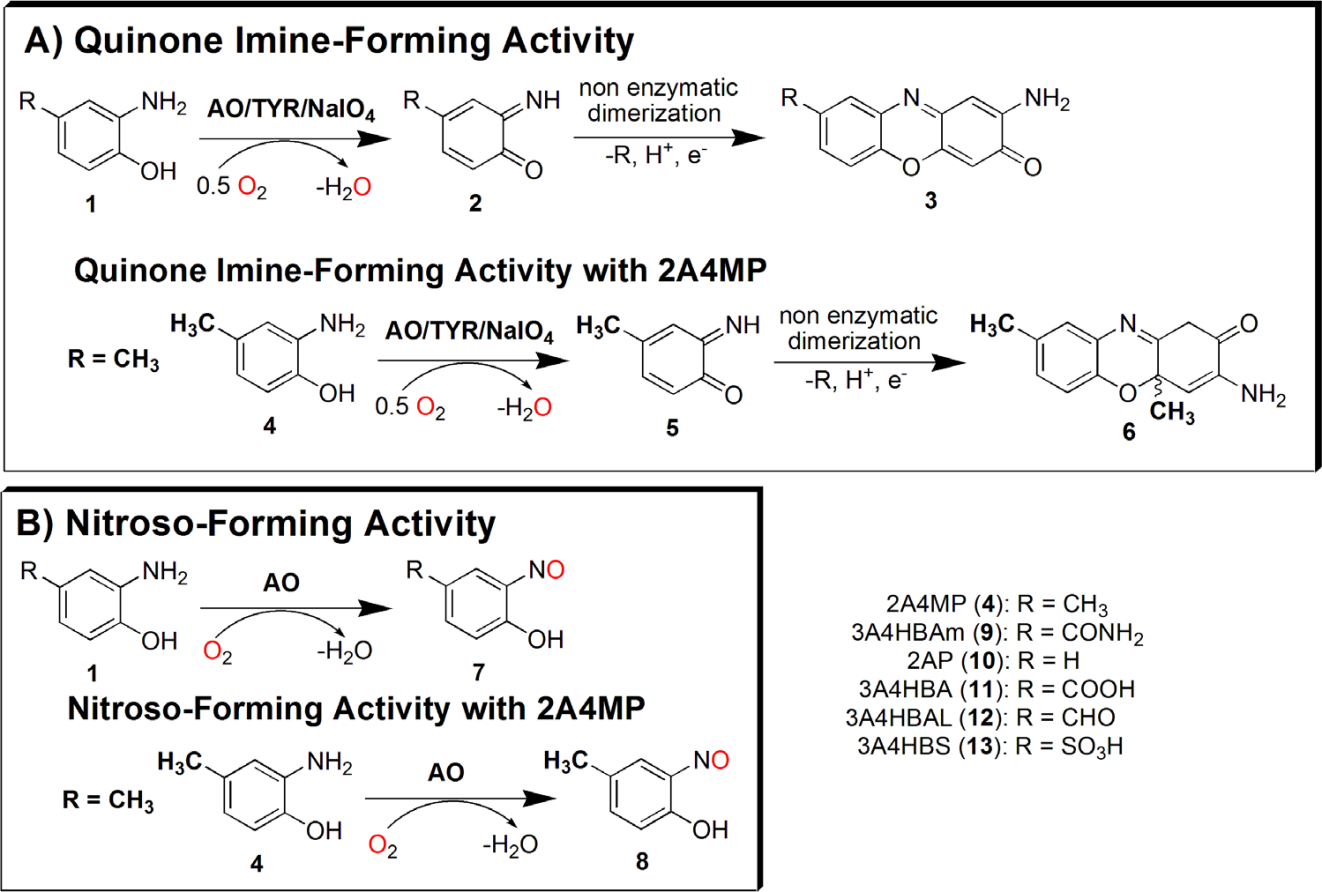
a) Oxidation of *o*‐aminophenols (**1**) by *o*‐aminophenol oxidase (AO), tyrosinase (TYR), or NaIO_4_ which results in the formation of quinone imines (**2**) and subsequent formation of phenoxazinones (**3**).^[^ ^9^, ^10^, ^11^, ^21^, ^22^ ^]^ When R is a methyl group, the compound 3‐amino‐1,4a‐dihydro‐14a,8‐dimethyl‐2H‐phenoxazin‐2‐one (APODM, **6**) forms after the production of the quinone imine intermediate (**2**).^[^ ^22^, ^23^ ^]^ b) Enzymatic oxidation of *o*‐aminophenols (**1**) at the amino group leads to the formation of nitrosophenols (**7**).^[^ ^3^, ^11^ ^]^ Structures of *o*‐aminophenol derivatives (R  =  *p*‐residue): 2‐amino‐4‐methylphenol (2A4MP, **4**) 3‐amino‐4‐hydroxy benzamide (3A4HBAm, **9**), *o*‐aminophenol (2AP, **10**), 3‐amino‐4‐hydroxybenzoic acid (3A4HBA, **11**), 3‐amino‐4‐hydroxybenzaldehyde (3A4HBAL, **12**) and 3‐amino‐4‐hydroxybenzenesulfonic acid (3A4HBS, **13**). The abbreviations of the substrates are chosen according to Noguchi et al., 2010.^[^ ^11^ ^]^

The activity ratios of the oxidation products formed by AOs (nitrosophenol (**7**, Figure 1: **reaction scheme B**) vs phenoxazinone (**3**, Figure 1: **reaction scheme A**)) depend on the steric and electron‐withdrawing/electron‐donating properties of the *p*‐residue of the *o*‐aminophenol substrate (Figure 1: R  =  *p*‐residue).^[^ ^11^ ^]^ In the case of 3‐amino‐4‐hydroxy benzamide (3A4HBAm, **9**, Figure 1), which constitutes a primary amide, only the formation of the nitroso product (**7**, Figure 1: **reaction scheme B**) is observed, while the enzymatic oxidation of *o*‐aminophenol (2AP, **10**, Figure 1), which lacks a steric *p*‐residue, leads exclusively to the formation of the phenoxazinone product (**3**, Figure 1: **reaction scheme A**).^[^ ^11^ ^]^ Based on the product formed, we conclude that a sterically demanding substituent may be necessary for the nitroso‐forming activity (Figure 1: **reaction scheme B**) to guide the substrate in the enzyme's active center as well as an inductive substituent to enhance the reactivity of the aromatic substrate. Furthermore, when 3‐amino‐4‐hydroxybenzoic acid (3A4HBA, **11**), 3‐amino‐4‐hydroxybenzaldehyde (3A4HBAL, **12**, Figure 1) and 3‐amino‐4‐hydroxybenzenesulfonic acid (3A4HBS, **13**, Figure 1) are enzymatically oxidized by AOs, both the phenoxazinone product and nitroso product are formed.^[^ ^11^ ^]^ 3A4HBA (**11**, Figure 1) and 3A4HBS (**13**, Figure 1) exhibit significantly lower nitroso‐forming activity (Figure 1: **reaction scheme B**) compared to 3A4HBAL (**12**, Figure 1) and 3A4HBAm (**9**, Figure 1).^[^ ^11^ ^]^ The decreased activity can be attributed to electron‐withdrawing functional groups (carboxylic and sulfonic acids) in the *p*‐position. This suggests that the ability of a functional group located in the *p*‐position to either donate or withdraw electrons governs the reactivity of a substrate. Up to date, all identified AOs are produced by bacteria that are phylogenetically classified as *Actinomycetes*, which are typically present in soil, water, and on plants.^[^ ^24^ ^]^ *Streptomyces* species, which form a genus within the bacterial order of *Actinomycetes*, are known for their versatile potential to produce bioactive compounds such as antibiotics, pigments, and melanoidins.^[^ ^25^, ^26^, ^27^ ^]^ Consequently, they are the source of therapeutically effective natural compounds such as the antibiotics chloramphenicol (Figure S2 (1)), grisemycin (Figure S2 (2)), and streptomycin (Figure S2 (3)).^[^ ^27^, ^28^ ^]^ The yellow pigment Grixazone B (Figure S2 (4)) present in *Streptomyces griseus* was reported to show parasiticidal activity.^[^ ^9^ ^]^ The AO from *Streptomyces griseus* (*Sg*GriF, Figure 2) which is, among other enzymes, involved in the Grixazone biosynthesis pathway, was recombinantly expressed and catalytically investigated previously.^[^ ^9^ ^]^ *Sg*GriF (35.74 kDa) is active towards *o*‐aminophenols (**1**, Figure 1) over a pH range from pH 6.0 to pH 11.0, with a maximum activity between pH 8.5 and pH 10.5, depending on the investigated substrate.^[^ ^9^ ^]^ Moreover, the optimal temperature at pH 7.0 for the enzymatic activity of *Sg*GriF is relatively high at 55 °C.^[^ ^9^ ^]^

**Figure 2:**
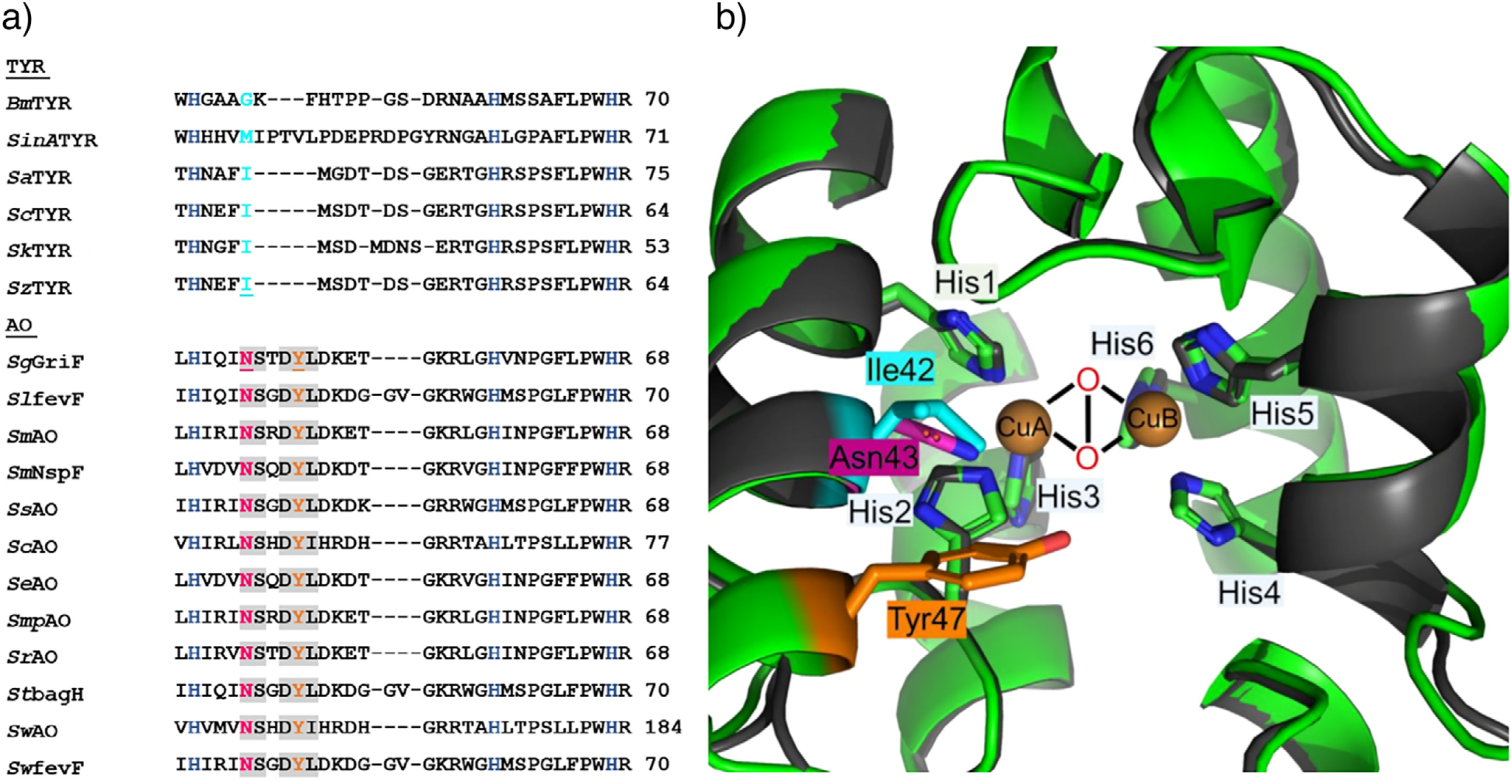
a) Sequence alignment of TYRs *Bm*TYR (PDB: 3NM8), *SinA*TYR (UniprotKB: L0D705), *Sa*TYR (PDB: 6J2U), *Sc*TYR (PDB: 1WX2), *Sk*TYR (UniprotKB: A0A077HD11), and *Sz*TYR (UniprotKB: A0A2S3Y8X7) with AOs *Sg*GriF (UniprotKB: B1VTI5), *Sl*fevF (UniprotKB: A0A2H5BVB1), *Sm*AO (UniprotKB: A0A1W7D1S4), *Sm*NspF (UniprotKB: D6RTB9), *Ss*AO (UniprotKB: A0A0W7WYA7), *Sc*AO (UniprotKB: A0A1Q4VUB3), *Se*AO (UniprotKB: A0A197SSE1), *Smp*AO (UniprotKB: A0A1V2RQ55), *Sr*AO (UniprotKB: A0A2S4YIE3), *St*bagH (UniprotKB: A0A482LS25), *Sw*AO (UniprotKB: A0A429IXZ5) and *Sw*fevF (UniprotKB: L0N6P7).^[^ ^29^ ^]^ Letters (dark blue): first, second, and third histidine coordinating copper A center. Letters (shaded in grey): conserved residues present only in AOs. Letters (magenta): conserved asparagine. Letters (orange): conserved tyrosine. Letters (cyan): residues in TYRs homologous to conserved asparagine in AOs. b) Structural alignment of the active center of *Sg*GriF (AlphaFoldDB model (B1VTI5)) cartooned in green and *Sz*TYR from *Streptomyces* sp. ZL‐24 (AlphaFoldDB model (A0A429IXZ5)) cartooned in dark grey.^[^ ^30^, ^31^ ^]^ Ile42 in *Sz*TYR is depicted in cyan. Conserved Asn43 in *Sg*GriF is shown in magenta, and conserved Tyr47 in *Sg*GriF is highlighted in orange. Histidines coordinating CuA and CuB are numbered from His1 to His6 starting from N‐terminus, and nitrogen atoms are depicted in blue and oxygens in red. Models are taken from AlphaFold DB^[^ ^30^, ^31^ ^]^ and visualized with the PyMOL Molecular Graphics System, Version 3.0 Schrödinger, LLC.

Up to date, the structural basis for the observed catalytic differences between TYRs (quinone imine‐forming activity; Figure 1: **reaction scheme A**) and AOs (nitroso‐forming activity; Figure 1: **reaction scheme B**) is still unresolved. Herein, one amino acid residue that is responsible for the nitroso‐forming activity in type‐III copper proteins is identified.

*Sequence and structural investigations*: The first sequence analysis aimed at identifying structural differences between AOs and TYRs was conducted by Ginsbach et al. (2012).^[^ ^3^ ^]^ They highlighted Asp46 and Met234 in the *o*‐aminophenol oxidase from *Streptomyces murayamaensis* (*Sm*NspF, Figure 2a) as unique residues distinguishing AOs from TYRs. Utilizing recent advances in next‐generation sequencing and protein structure prediction, we were able to perform a broader sequence and confident structural alignment to identify the amino acid positions responsible for the different enzymatic behaviors of TYRs and AOs.^[^ ^30^, ^31^ ^]^ A sequence alignment was conducted by comparing bacterial AOs, which are phylogenetically closely related to *Sg*GriF, with well‐characterized bacterial TYRs from various phyla (Figure 2a). The bacterial TYRs from *Streptomyces castaneoglobisporus*, *Streptomyces avermitilis*, and *Bacillus megaterium* are well qualified for structural comparisons since crystallographic data are available.^[^ ^32^, ^33^, ^34^ ^]^ In addition, TYRs from *Singulisphaera acidiphila*, *Streptomyces kathirae*, and *Streptomyces* sp. ZL‐24 were chosen to elucidate the structural differences between TYRs and AOs. These six bacterial TYRs are qualified for structure alignments and subsequent mutagenesis studies due to the availability of well‐established expression protocols.^[^ ^34^, ^35^, ^36^ ^]^ The sequence and structure alignment identified two highly conserved residues that are only present in the vicinity of the active sites of AOs (Figure 2a, orange) and which are located at the surface of the substrate binding pocket (Figures 2b and S3).^[^ ^30^, ^31^ ^]^ The localization of the side chain of the conserved tyrosine residue (Y, Tyr47 in *Sg*GriF) in AOs is a striking difference as structural alignments reveal the homologous location to be unoccupied in all six investigated TYRs (Figures 2, S4, and S5). The second highly conserved amino acid residue is an asparagine (N, Asn43 in *Sg*GriF) in AOs (Figure 2a, magenta), which is not conserved in TYRs (Figure 2a, cyan). At the position of the highly conserved asparagine residue (Figure 2b, magenta), which is present in all investigated AOs, TYRs tend to have a non‐polar and aliphatic amino acid at the homologous position (Figure 2a, cyan). Interestingly, all investigated TYRs from *Streptomyces* species exhibit a non‐polar and aliphatic isoleucine (Figure 2a). Both tyrosine and asparagine side chains are suitable hydrogen bond acceptors that may interact with the substrate's hydroxy group as hydrogen bond donors and can, therefore, be suspected to impact the catalytic behavior of the AOs. For TYRs, asparagine residues located in proximity to the dicopper center of type‐III copper proteins have been proposed to control tyrosinase activity by acting as proton shuttles and by tuning the basicity of the copper coordinating histidines.^[^ ^6^ ^]^

*Mutations of SgGriF and SzTYR*: To test the impact of the highly conserved tyrosine residue in AOs (Y, Tyr47 in *Sg*GriF), which is absent in TYRs, Tyr47 in *Sg*GriF was mutated to a small, hydrophobic valine residue (see Supporting Information: Experimental Procedures 1.3). Valine was chosen as it, contrary to the tyrosine residue, features a non‐polar and sterically small side chain. Additionally, a second *Sg*GriF mutant was designed by replacing the asparagine residue highly conserved in AOs with isoleucine, which naturally occurs at the homologous position in wild‐type tyrosinases from *Streptomyces* species. Alongside wild‐type *Sg*GriF, the two mutants *Sg*GriF‐Y47V and *Sg*GriF‐N43I were heterologously expressed. These mutants were designed to eliminate the nitroso‐forming activity (Figure 1: **reaction scheme B**) inherent to *Sg*GriF. Conversely, a mutant TYR was designed to induce nitroso‐forming activity in *Sz*TYR (*Streptomyces* sp. ZL‐24, Figure 2), which, in its wild‐type form, lacks this activity (see Supporting Information: Experimental Procedures 1.4). *Sz*TYR was chosen as a model enzyme due to its high sequence similarity (58.3%, alignment with Clustal Omega) to *Sg*GriF, with both enzymes originating from *Streptomyces* species.^[^ ^37^ ^]^ Additionally, its expression is supported by a well‐established protocol.^[^ ^33^ ^]^ To mimic the conserved asparagine residue in AOs (Figure 2a), the homologous Ile42 residue in *Sz*TYR (Figure 2a) was mutated to asparagine, resulting in *Sz*TYR‐I42N. The wild‐type and mutant enzymes were successfully expressed and purified, as shown in Figures S6 and S7, and further confirmed by SDS‐PAGE (Figure S8) and ESI‐MS (Figures S9–S13 and Tables 1 and S2) using protocols developed and optimized for each of the five enzymes based on published methods (see Supporting Information: Experimental Procedures 1.3, 1.4).^[^ ^36^, ^38^ ^]^

**Table 1:** Enzyme reaction rates and ESI mass spectra of recombinant AOs and TYRs. (**6**).

Enzyme	Calculated Mass (Da)	Experimental Mass (Da)	Spec. Act. at 400 nm (U mg^−1^)^a)^, ^b)^	Spec. Act. at 340 nm (U mg^−1^)^a)^, ^c)^	Activity Ratio 340 nm/400 nm
SgGriF	35737.28^d)^	35736.51 ± 0.96 (Figure S9)	7.81 ± 0.17	3.59 ± 0.08	0.46
*Sg*GriF‐Y47V	35673.17^d)^	35672.17 ± 1.37 (Figure S10)	2.96 ± 0.03	0.78 ± 0.05	0.26
*Sg*GriF‐N43I	35736.36^d)^	35735.64 ± 1.03 (Figure S11)	0.55 ± 0.02	n. d.	n. d.
*Sz*TYR	30892.59^d)^	30892.60 ± 1.20 (Figure S12)	0.60 ± 0.14	n. d.	n. d.
*Sz*TYR‐I42N	30893.53^d)^	30893.48 ± 0.99 (Figure S13)	0.090 ± 0.013	0.021 ± 0.005	0.23

^a)^ 1 Unit (U) = 1 µmol min^−1^.

^b)^ Quinone imine‐forming activity at 400 nm.

^c)^ Nitroso‐forming activity at 340 nm.

^d)^ The calculated mass was determined based on the protein sequence (UniprotKB *Sg*GriF: B1VTI5, UniprotKB *Sz*TYR: A0A2S3Y8X7).^[^ ^29^ ^]^ The experimental mass was determined by deconvolution based on at least 15 consecutive peaks located around the most intense charge state (see SI: section 1.6). Specific activities of enzymatic oxidation of 2A4MP (**4**) are reported, and “n. d.” indicates that the specific activities were not reliably determined due to low or missing activity. The corresponding first standard deviations are given after the ± sign. The experimental setup, including the calculation of the extinction coefficient and spec. act., is described in detail in the Supporting Information: Figure SA and section 1.8.

*Kinetic investigations*: The activity ratios of the oxidation products formed by AOs and TYRs were determined spectrophotometrically using the substrate 2A4MP (**4**, Figure 1) as it allows to effectively differentiate between quinone imine‐forming activity (Figure 1: **reaction scheme A**) at 400 nm and nitroso‐forming activity (Figure 1: **reaction scheme B**) at 340 nm. This differentiation is possible due to the characteristic and well‐separated absorption bands of the respective products (Figure SA).^[^ ^23^, ^39^, ^40^, ^41^ ^]^ The non‐enzymatic oxidation of 2A4MP (**4**, Figure 1) with sodium periodate (Figure 3a, black curve) and its enzymatic oxidation by wild‐type TYRs (Figure 3b, cyan curve) both exhibit a purely quinone imine‐forming character, resulting in the phenoxazinone reaction product (Figure 1: **reaction scheme A**). This oxidation product is spectrophotometrically observed as a single absorption band at 400 nm (Figure 3).^[^ ^23^ ^]^ On the contrary, an additional absorption band occurs at 340 nm, corresponding to the nitrosophenol product,^[^ ^39^, ^40^, ^41^ ^]^ when 2A4MP (**4**, Figure 1) is enzymatically oxidized by AOs. This indicates the simultaneous presence of quinone imine‐forming activity (Figure 1: **reaction scheme A**) and nitroso‐forming activity (Figure 1: **reaction scheme B**), the latter being exclusively performed by AOs. Accordingly, the enzymatic conversion of 2A4MP (**4**, Figure 1) by wild‐type *Sg*GriF (Figure 3a, orange curve) leads to the formation of two absorption bands at 340 and 400 nm, respectively. Despite a reduction in specific activity values (2.6‐fold lower at 400 nm and 4.6‐fold lower at 340 nm compared to the wild‐type, Table 1), the mutant enzyme *Sg*GriF‐Y47 V exhibits an absorption pattern similar to that of wild‐type *Sg*GriF (Figure 3a, red curve), with absorption bands at 340 and 400 nm during the enzymatic oxidation of 2A4MP (**4**, Figure 1). In contrast, the mutant enzyme *Sg*GriF‐N43I shows a complete loss of the nitroso‐forming activity (Figure 3a, purple curve), as no visible absorption band is formed at 340 nm, exhibiting reaction behavior characteristic for wild‐type TYRs (Figure 3b, cyan curve). Besides, it also shows a decrease in quinone imine‐forming activity (14.2‐fold lower at 400 nm, compared to the wild‐type, Table 1, Figure 1: **reaction scheme A**). The data presented herein demonstrate that a single mutation of a polar asparagine (Asn43) to a nonpolar isoleucine results in the knock‐out of the nitroso‐forming activity (Figure 1: **reaction scheme B**) in wild‐type *Sg*GriF, while the enzymatic quinone imine‐forming activity (Figure 1: **reaction scheme A**) is retained (Table 1), thereby converting an AO into a TYR.

**Figure 3:**
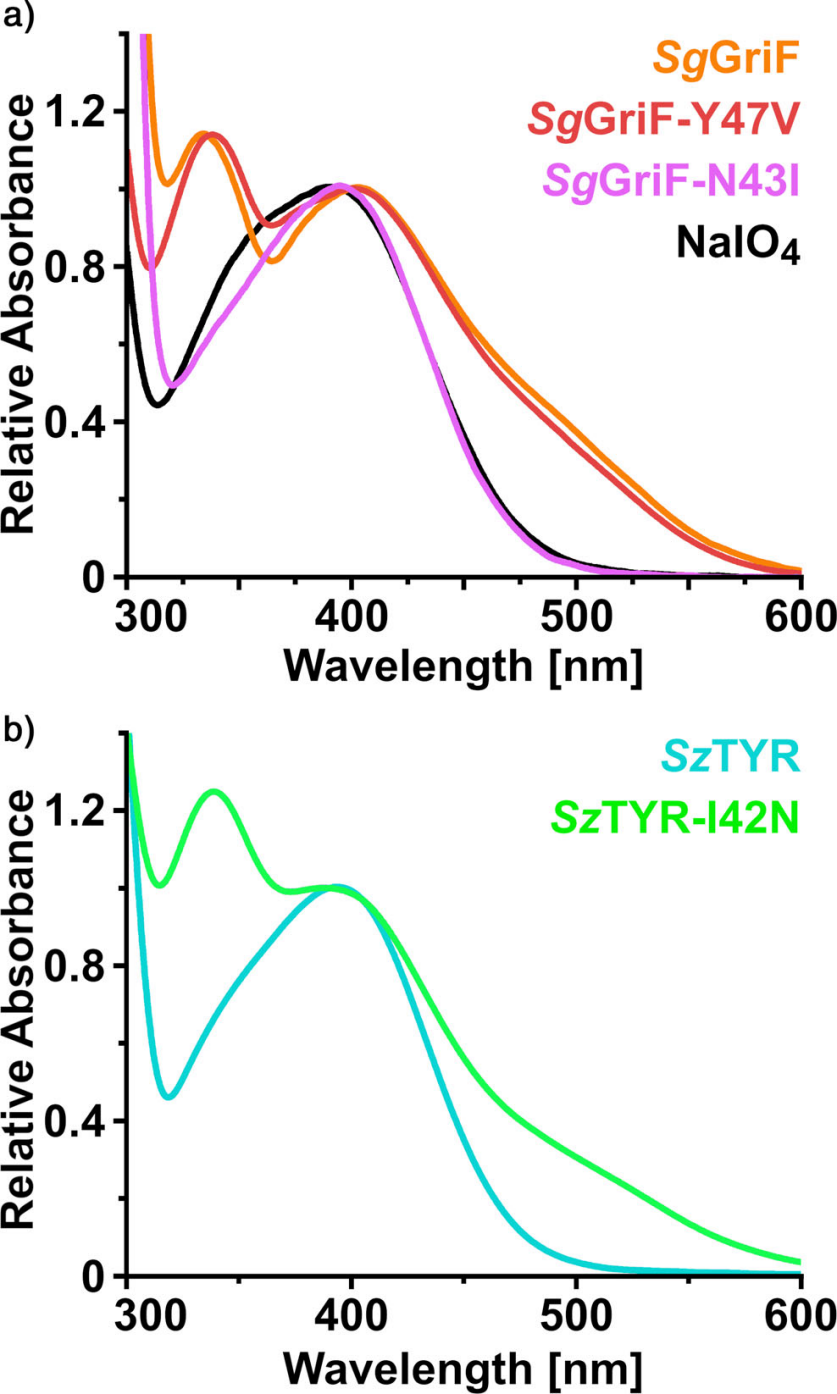
a) UV‐VIS spectra after formation of oxidation products at pH 7.5 by addition of sodium periodate (black curve), *Sg*GriF (orange curve), *Sg*GriF‐Y47 V (red curve), and *Sg*GriF‐N43I (purple curve) to 2A4MP (**4**). b) UV‐vis spectra after formation of oxidation products by addition of *Sz*TYR (cyan curve) and *Sz*TYR‐I42N (green curve) to 2A4MP (**4**). The experimental setup is described in detail in the Supporting Information (1.9 Evolution of oxidized 2A4MP and Figure S14).

When wild‐type *Sz*TYR catalyzes the oxidation of 2A4MP (**4**, Figure 1), a single absorption band at 400 nm appears, indicating the formation of APODM (**6**, Figure 1: **reaction scheme A**), with no visible evidence of nitroso‐product‐formation at 340 nm (Figure 3b, cyan curve). To test the hypothesis that Asn43 in *Sg*GriF governs the nitroso‐forming activity (Figure 1: **reaction scheme B**), we investigated the possibility of converting a TYR into an AO by targeting the homologous amino acid position (Ile42) in *Sz*TYR. The mutant *Sz*TYR‐I42N, which structurally resembles AOs such as *Sg*GriF at the mutated position, exhibits, in addition to product formation at 400 nm (quinone imine forming activity), a pronounced absorption band at 340 nm (nitroso‐forming activity, Figure 3b, green curve). Activity values for *Sz*TYR‐I42N (Table 1) demonstrate an activity ratio (nitroso‐forming activity vs quinone imine‐forming activity) comparable to that of AOs (*Sz*TYR‐I42N: 0.23, *Sg*GriF: 0.46, *Sg*GriF‐Y47V: 0.25). These findings indicate that the nitroso‐forming activity (Figure 1: **reaction scheme B**), which is absent in wild‐type *Sz*TYR, is present in the *Sz*TYR‐I42N mutant.

Interactions between amino acid side chains and adjacent copper coordinating histidines have been reported to control their basicity and their ability to engage in substrate proton shuffling, which governs their catalytic performance.^[^ ^6^ ^]^ This is further substantiated by experimental evidence presented herein, as Asn43/Ile42 (termed activity selector) is located adjacent to the second CuA coordinating histidine (Figure 2b). To investigate the structural basis for the observed catalytic differences in *Sg*GriF and *Sz*TYR in‐depth, molecular docking studies have been performed and revealed different orientations of 2A4MP (**4**, Figure 1), depending on the presence or absence of an asparagine in the activity selector position. In *Sg*GriF, the amide group of Asn43 engages in H‐bonding with the phenolic hydroxy group of 2A4MP which leaves the amino group of 2A4MP, exposed towards the dicopper center (Figure 4a). In contrast, in *Sz*TYR the phenolic hydroxy group is oriented towards the dicopper center (Figure 4b), as no H‐bonding is present due to the presence of an isoleucine (Ile42). Thus, it can be hypothesized that direct interactions with the substrate as well as interactions with adjacent copper coordinating histidines explain the critical importance of the amino acid residue featured in the activity selector position.

**Figure 4:**
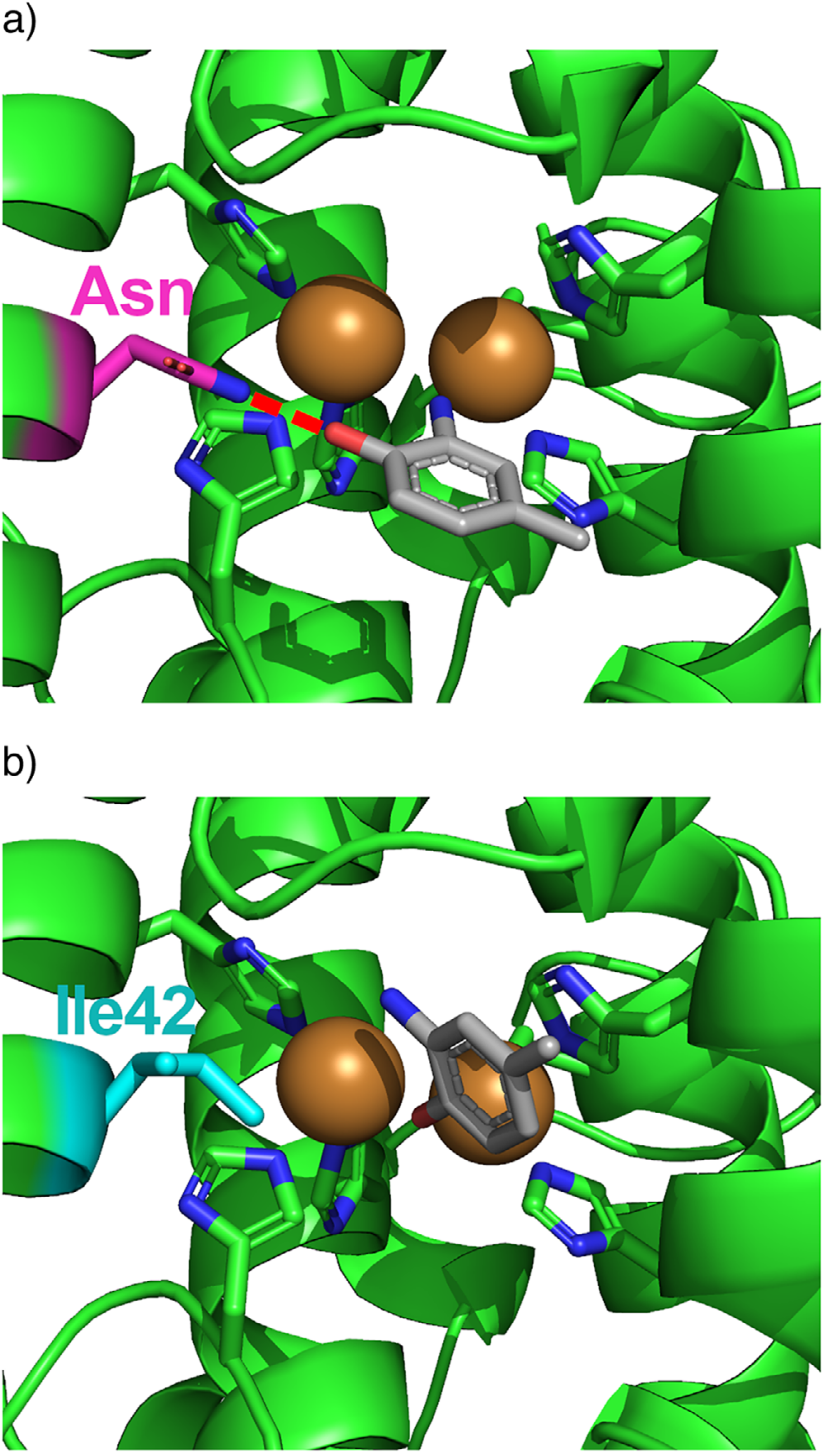
Docking poses of 2A4MP (**4**, Figure 1) in the active centers of *Sg*GriF a) and *Sz*TYR b): a) In *Sg*GriF, the phenolic hydroxy group of 2A4MP (grey) is stabilized by H‐bonding (red dotted line, distance: 2.2 Å) to the amide group of asparagine (Asn43, magenta). This leads to the nitrogen atom from the amino group of 2A4MP being oriented towards the dicopper center. b) As no stabilizing H‐bonding is present in *Sz*TYR, which features an isoleucine (Ile42, cyan) instead of an asparagine in the homologous position, the hydroxy group of 2A4MP (grey) is oriented towards the dicopper center in *Sz*TYR, which provides a structural explanation for the different reactivities of *Sg*GriF and *Sz*TYR.

In summary, the structural differences underlying the catalytic behaviors of two bacterial type‐III copper enzymes, TYRs and AOs, have been investigated. While both enzymes exhibit quinone‐imine forming activity, AOs uniquely perform nitroso‐forming activity. It is demonstrated that the amino acid located four positions after the first histidine coordinating the copper A center (Asn43 in *Sg*GriF, Ile42 in *Sz*TYR) functions as an activity selector, governing the nitroso‐forming activity in AOs. By analyzing sequence and structural data, highly conserved amino acid positions in the catalytic center of AOs have been identified, and mutagenesis studies have been conducted to successfully convert a TYR into an AO and vice versa. The ability to oxidize various small phenolic compounds by controlling the nitroso‐forming activity in enzymatic oxidations will profoundly impact a broad range of applications in the fields of medicinal and organic chemistry.^[^ ^26^, ^42^ ^]^

## Supporting Information

The authors have cited additional references within the Supporting Information.^[^ ^42^, ^43^, ^44^, ^45^, ^46^, ^47^, ^48^, ^49^, ^50^ ^]^

## Conflict of Interests

The authors declare no conflict of interest.

## Supporting information



Supporting Information

## Data Availability

The data that support the findings of this study are openly available in phaidra at https://phaidra.univie.ac.at/detail/o:2119942.
