# Supplementary material for: Identification of an Activity Selector for the Nitroso‐Forming Activity in Bacterial Type‐III Copper Enzymes
Source: Angew Chem Int Ed Engl. 2025 May 24;64(26):e202501560. doi: 10.1002/anie.202501560 (PMC12184298; doi:10.1002/anie.202501560)
Supplement: Supplementary file 1 — Supporting Information [file ANIE-64-e202501560-s001.docx]

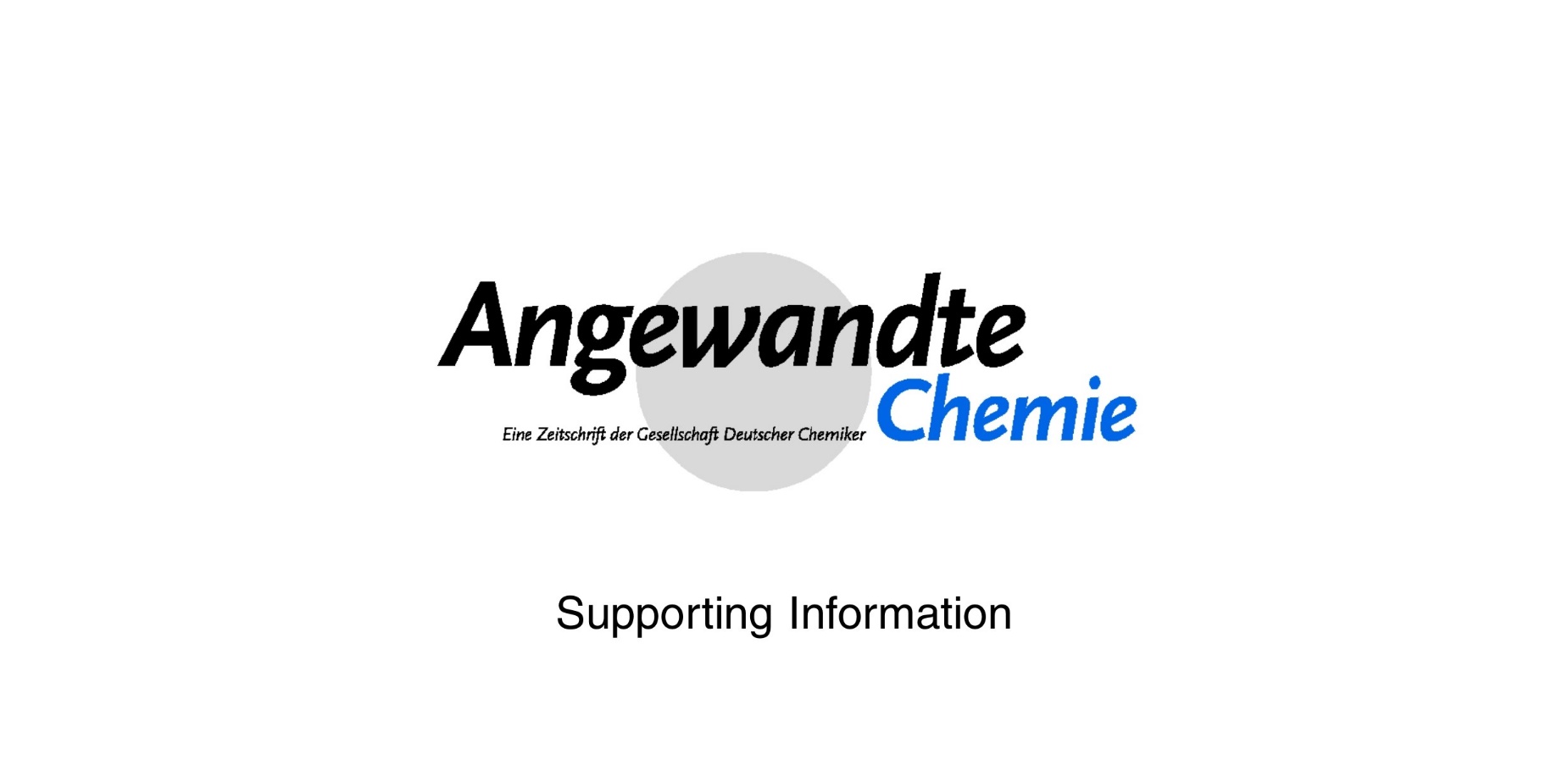


Supporting Information

**Identification of an Activity Selector for the Nitroso-Forming Activity in Bacterial Type-III Copper Enzymes**

*Hoa Le Xuan^[a,b]^, Felix Panis^[a]^ and Annette Rompel*^[a,b]^*

[a] H. Le Xuan, M.Sc., Dr. F. Panis, A. Rompel

Universität Wien, Fakultät für Chemie,

Institut für Biophysikalische Chemie,

Josef-Holaubek Platz 2, 1090 Vienna (Austria),

E-mail: [annette.rompel@univie.ac.at](mailto:annette.rompel@univie.ac.at)

Homepage: <https://www.bpc.univie.ac.at>

[b] H. Le Xuan, M.Sc., A. Rompel

Vienna Doctoral School in Chemistry (DoSChem),

Universität Wien,

Währingerstraße 42, 1090 Vienna (Austria)

DOI: 10.1002/anie.2021XXXXX

**Author Contributions**

H. L. X. Conceptualization: Lead; Data curation: Lead; Writing - Review & Editing: Lead

F. P. Conceptualization: Supporting; Data curation: Supporting; Writing - Review & Editing: Supporting

A. R. Funding acquisition: Lead; Project administration: Lead; Supervision: Lead; Writing - Review & Editing: Supporting

Table of Contents

[**Author Contributions** 2](#_Toc191377592)

[List of abbreviations 4](#_Toc191377593)

[1. Experimental Procedures 6](#_Toc191377594)

[1.1 Cloning and mutations around the catalytic dicopper center of SgGriF 6](#_Toc191377595)

[1.2 Mutations around the catalytic dicopper center of SzTYR 6](#_Toc191377596)

[1.3 Heterologous expression in E. coli and purification of SgGriF and SgGriF-mutants 6](#_Toc191377597)

[1.4 Heterologous expression in E. coli and purification of SzTYR and SzTYR-I42N 8](#_Toc191377598)

[1.5 SDS-PAGE analysis 8](#_Toc191377599)

[1.6 Intact protein mass spectrometry 8](#_Toc191377600)

[1.7 Determination of extinction coefficients 9](#_Toc191377601)

[1.8 Substrate activity assays 10](#_Toc191377602)

[1.9 Evolution of oxidized 2A4MP 11](#_Toc191377603)

[1.10 Docking Experiments. 11](#_Toc191377604)

[2. Results 11](#_Toc191377605)

[2.1. Supplementary Figures 11](#_Toc191377606)

[Figure S1 Monophenolase and diphenolase activity 11](#_Toc191377607)

[Figure S2 Bioactive substances 12](#_Toc191377608)

[Figure S3 Binding pocket of SgGriF 12](#_Toc191377609)

[Figure S4 Structural alignments of AOs 13](#_Toc191377610)

[Figure S5 Structural alignments of TYRs 14](#_Toc191377611)

[Figure S6 Purification of SgGriF and SgGriF-Y47V 15](#_Toc191377612)

[Figure S7 Purification of SgGriF-N43I, SzTYR and SzTYR-I42N 16](#_Toc191377613)

[Figure S8 Reducing SDS-PAGE (13 %, untruncated) of recombinant proteins 17](#_Toc191377614)

[Figure S9 ESI-MS of SgGriF 17](#_Toc191377615)

[Figure S10 ESI-MS of SgGriF-Y47V 18](#_Toc191377616)

[Figure S11 ESI-MS of SgGriF-N43I 19](#_Toc191377617)

[Figure S12 ESI-MS of SzTYR 20](#_Toc191377618)

[Figure S13 ESI-MS of SzTYR-I42N 21](#_Toc191377619)

[Figure S14 Evolution of oxidized 2A4MP absorption spectra in TRIS-HCl buffer 22](#_Toc191377620)

[2.2 Supplementary Tables 23](#_Toc191377621)

[Table S1 Designed primers with annealing temperatures. 23](#_Toc191377622)

[Table S2 Purification of expressed enzymes. 23](#_Toc191377623)

[SzTYR-I42N 24](#_Toc191377624)

[3 Data Management Plan 25](#_Toc191377625)

[4 References 25](#_Toc191377626)

# List of abbreviations

**×g** times gravity

**2A4MP** 2-amino-4-methylphenol

**2AP** 2-aminophenol

**3A4HBA** 3-amino-4-hydroxybenzoic acid

**3A4HBAL** 3-amino-4-hydroxybenzaldehyde

**3A4HBAm** 3-amino-4-hydroxy benzamide

**3A4HBS** 3-amino-4-hydroxybenzenesulfonic acid

**4M2NP** 4-methyl-2-nitrosophenol

**AC** affinity chromatography

**AEX** anion exchange chromatography

**AO** *o*-aminophenol oxidase

**APODM** 3-amino-1,4a-dihydro-14a,8-dimethyl-2*H*-phenoxazin-2-one

**Asn** asparagine

**ATCC** American Type Culture Collection

**ATP** adenosine triphosphate

**CO** catechol oxidase

**DE3** λDE3 lysogen (prophage carrying the T7 RNA polymerase gene under control of the lacUV5 promoter)

**DNA** deoxyribonucleic acid

**DSMZ** Deutsche Sammlung von Mikroorganismen und Zellkulturen

**E** elution step

***E. coli*** *Escherichia coli*

***Ec*GroL** *Escherichia coli* GroEL chaperonin

**EDTA** ethylenediaminetetraacetic acid

**ESI** Electrospray ionization

**FT** flow through

**fwd** forward

**GE** General Electric

**Gly** glycine

**GSH** reduced glutathione

**GST** glutathione S-transferase

**GSTrap FF** Glutathione Sepharose Fast Flow column

**h** hours

**HRV3C** human rhinovirus (serotype 14B) protease 3C

**Ile** isoleucine

**IPTG** isopropyl β-D-1-thiogalactopyranoside

**LB** lysogeny broth: 10 gl^-1^ peptone or tryptone, 5 gl^-1^ yeast extract and 10 gl^-1^ NaCl in water

**M** molar; mole per liter; in pseudomolecular ions: mass

**Met** methionine

**MS** mass spectrometry

**n. d.** not determined

**NL** normalization level

**mAU** milli absorption units; 1 mAU corresponds to an absorbance of 0.001 cm^-1^.

**NEB** New England Biolabs

**OD_600_** optical density at a wavelength of 600 nm

**PAGE** polyacrylamid gel electrophoresis

**PCR** polymerase chain reaction

**RT** room temperature

**rev** reverse

**rpm** revolutions per minute

**S/N** signal-to-noise ratio

**SDS** sodium dodecyl sulfate; sodium lauryl sulfate

**SEC** size exclusion chromatography

***Sg*GriF** *o*-aminophenol oxidase originating from *Streptomyces griseus*

***Sg*GriF-N43I** *o*-aminophenol oxidase originating from *Streptomyces griseus* with replacement of asparagine 43 by isoleucine

***Sg*GriF-*Y47V*** *o*-aminophenol oxidase originating from *Streptomyces griseus* with replacement of tyrosine 47 by valine

**SI** supporting information

***SinA*TYR** tyrosinase originating from *Singulisphaera acidiphila*

**Spec. Act.** specific activity

***Sz*TYR** tyrosinase originating from *Streptomyces* sp. ZL-24

***Sz*TYR-I42N** tyrosinase originating from *Streptomyces* sp. ZL-24 with replacement of isoleucine 42 by asparagine

**T** temperature

**TRIS** tris(hydroxymethyl)aminomethane; 2-amino-2-(hydroxymethyl)propane-1,3-diol

**TYR** tyrosinase (EC 1.14.18.1)

**Tyr** tyrosine

**U** unit (of enzymatic activity); 1 U = 1 μmolmin^-1^ of transformed substrate (2A4MP, **Figure 1** (**4**))

**W** washing step

# Experimental Procedures

1.1 Cloning and mutations around the catalytic dicopper center of SgGriF**.** The gene encoding *Sg*GriF was amplified from *Streptomyces griseus* (DSMZ, Braunschweig, Germany) by PCR with Q5 High-Fidelity DNA polymerase (NEB: New England Biolabs; Ipswich, MA, USA) according to the supplier’s protocol using *Sg*GriF-FWD and *Sg*GriF-REV as primers (Table S1). The resulting PCR-product was cloned into the pGEX-6P-SG-vector using Esp3I (NEB, recognition site: 5´…CGTCTC(N)_1_|…3´, 3´…GCAGAG(N)_5_|…5´) cutting sites and T4 DNA ligase (NEB) simultaneously at 30 °C for 90 min according to the supplier’s protocol. The ligased vector carrying the *Sg*GriF-gene was used as template for the construction of the mutants *Sg*GriF-Y47V and *Sg*GriF-N43I using the following primers: *Sg*GriF-Y47V-FWD, *Sg*GriF-Y47V-REV, *Sg*GriF*-*N43I-FWD and *Sg*GriF-N43I-REV (Table S1). The resulting linear PCR product was phosphorylated with T4 polynucleotide kinase (NEB) and was cyclized with T4 DNA ligase (NEB) simultaneously at 30 °C for 90 min. All cloned vectors were sequenced to verify the correctness of the inserted gene. Heterologous expression was performed after transforming sequence-verified vectors into *E. coli* BL21 (DE3) cells.

1.2 Mutations around the catalytic dicopper center of SzTYR**.** Cloning of *Sz*TYR in the pGEX-6P vector has been described by Panis *et al.*, 2021.^1^ The pGEX-6P vector carrying the *Sz*TYR-gene was used as a template for the construction of the mutant *Sz*TYR-I42N using the following primers: *Sz*TYR-I42N-FWD, *SzTYR*-I42N-REV (Table S1). The resulting linear PCR product was phosphorylated with T4 polynucleotide kinase (NEB) and was cyclized with T4 DNA ligase (NEB) simultaneously at 30 °C for 90 min. Heterologous expression was performed after transformation of sequence-verified vectors into *E. coli* BL21 (DE3) cells.

1.3 Heterologous expression in E. coli and purification of SgGriF and SgGriF-mutants**.** The enzymes (*Sg*GriF, *Sg*GriF-Y47V and *Sg*GriF*-*N43I) were N-terminally fused with the Glutathione-S-Transferase (GST) tag of the pGEX-6P-vector under the control of separate tac-promotors and lac-operator. The human rhinovirus 3C protease (HRV3C) recognition sequence (LEVLFQ|GP) was located between the fusion partners enabling enzymatic cleavage. The heterologous expression was performed with auto-induction medium (5.0 g/l yeast extract, 10.0 g/l tryptone, 2.0 mM MgSO_4_, 500.0 mM NaCl, 25.0 mM Na_2_HPO_4_, 25.0 mM KH_2_PO_4_, 50.0 mM NH_4_Cl, 5.0 mM Na_2_SO_4_, 5.0 g/l glycerol, 0.5 g/l D-glucose, and 2.0 g/l *α*-D-lactose) supplemented with 100 µg/ml ampicillin. Saturated overnight cultures of *E. coli BL21* (DE3) along with the vector that had been included were added to the auto-inducing medium to a starting OD_600_ of 0.02, and the mixtures were then shaken 230 rpm at 37 °C till the OD_600_ reached a value between 0.50 and 0.60. The cell cultures were incubated for 24 hours at 230 rpm and 15 °C. Afterwards, 2.0 mM CuSO_4_ were added to the auto-induction media, which were shaken 230 rpm at 15 °C for 24 hours. The cells were harvested by centrifugation of the expression media at 6000 ×g for 20 min at 4 °C. The supernatants were discarded, and the pellets were resuspended in lysis buffer (20 mM sodium phosphate, 300 mM NaCl, and 1 mM EDTA at pH 7.4) supplemented with 0.5 g/l lysozyme, 1 mM phenylmethylsulfonyl fluoride and 1 mM benzamidine. Subsequently, the cell suspensions were incubated for 45 min at 4 °C. The freeze-thaw technique was performed five consecutive times using liquid nitrogen and a water bath at 25 °C. Afterwards, 2 mM MgCl_2_ and 0.02 g/l DNase I were added, and the mixtures were incubated at 25 °C for 15 min at 200 rpm. The resulting suspensions were centrifuged at 8000 ×g for 60 min at 4 °C. The pellets were discarded, and the supernatants were filtrated before executing the purification steps using an ÄKTA Purifier (GE; Boston, MA, USA) placed in a refrigerator at 4 °C. The first purification step of the fusion proteins was carried out using a 5 ml GSTrap FF (GE) affinity column and a binding buffer (50 mM TRIS-HCl and 200 mM NaCl, pH 8.1). After a washing step (WS) using 60 ml washing buffer (20 mM TRIS, 300 mM KCl, 10 mM MgCl_2_, 5 mM ATP, pH 8.0), the trapped GST-tagged proteins were eluted and collected using binding buffer supplemented with 15 mM reduced glutathione during the elution step (E) (**Figure S6, S7**). The fusion proteins were concentrated and buffer exchanged to pure binding buffer using a Vivaspin ultrafiltration device (Sartorius, Göttingen, Germany) with a 30 kDa molecular weight cut-off. The GST-tags were removed by incubating the fusion protein samples with GST-HRV3C, which was produced in-house^2^, overnight at 4 °C with a mass ratio of 1:50 (protease:fusion protein). Then, for the second purification step the sample mixture of GST-HRV3C protease and cleaved fusion protein was purified using a 5 ml GSTrap FF (GE) affinity column and the binding buffer (**Figure S6, S7**). GST tags and the GST-tagged protease were trapped by the column while the target proteins passed through the column and were collected. The target proteins were again concentrated using the Vivaspin ultrafiltration device (Sartorius) with a 30 kDa molecular weight cut-off and the buffer was exchanged to sodium-citrate buffer (40 mM trisodiumcitrate, 10 mM citric acid, pH 7.1). To remove remaining impurities such as the *Ec*GroL chaperon produced from *E. coli* and attached to the recombinant proteins, the enzymes were purified by size exclusion chromatography (SEC) using a Superdex 75 column (GE) (**Figure S6, S7**).^3^ The SEC was carried out with sodium-citrate buffer as running buffer. Subsequently, the peak fraction was collected and concentrated using the Vivaspin ultrafiltration device (Sartorious) with a 30 kDa molecular weight cut-off. The buffer was then exchanged to the binding buffer (50 mM TRIS-HCl and 200 mM NaCl, pH 8.1) and enzymes were concentrated and stored at 4 °C. Protein concentrations were determined according to the Lambert-Beer law using the extinction coefficient (*Sg*GriF: 79870 M^-1^cm^-1^, *Sg*GriF-Y47V: 78380 M^-1^cm^-1^, *Sg*GriF*-*N43I: 79870 M^-1^cm^-1^) calculated by ExPASy ProtParam at 280 nm.^4,5^ The purity of the recombinantly expressed proteins was analyzed using 13% SDS-PAGE (**Figure S8**).

1.4 Heterologous expression in E. coli and purification of SzTYR and SzTYR-I42N**.** The expression protocol has been described in detail previously by Panis *et al.*, 2021.^1^ In short, the pGEX-6P expression vector carrying the SzTYR/SzTYR-I42N-gene and the codon-optimized version of the caddie protein A0A2S3Y8X5, both under the control of separate tac-promotors and lac-operators, was transformed into *E. coli* BL21(DE3). Saturated overnight cultures of *E.* coli BL21 (DE3) along with the included vector were added to the LB medium (10 g/L tryptone, 10 g/L NaCl, 2.0 mM MgSO_4_ and 5 g/L yeast extract) supplemented with 100 µg/ml ampicillin and incubated at 37 °C and 230 rpm until an OD_600_ value of 0.5−0.6 was reached. Subsequently, 0.5 mM isopropyl-*β*-D-thiogalactopyranoside (IPTG) and 0.5 mM CuSO_4_ were added, and the expression medium was incubated at 19 °C for 60 h at 230 rpm. The expression batch was centrifuged for 20 min at 6000 ×g, and the supernatant was precipitated by adding 262 g/l (NH_4_)_2_SO_4_ (45% saturation) and incubated at 4 °C for 45 min. The pellet was obtained by centrifuging at a speed of 8 000 ×g for 15 min at 4 °C, and then it was resuspended in 10 mM TRIS-HCl at pH 7.5. A Vivaspin ultraﬁltration device with molecular weight cutoﬀ of 30 kDa (Sartorius) was used to exchange the buffer to 10 mM TRIS-HCl at pH 7.5. Afterwards, the sample was injected to a MonoQ anion exchange column (GE) in 10 mM TRIS−HCl at pH 7.5 (**Figure S7**). The target enzyme was eluted without binding to the column. Protein concentrations were determined according to the Lambert-Beer law using the extinction coefficient (*Sz*TYR: 74940 M^-1^cm^-1^, *Sz*TYR-I42N: 74940 M^-1^cm^-1^) calculated by ExPASy ProtParam at 280 nm.^4,5^ The purity of the recombinantly expressed proteins was analyzed using 13% SDS-PAGE (**Figure S8**). Enzymes were stored in 10 mM TRIS-HCl at pH 7.5 at 4 °C and used immediately for further experiments.

1.5 SDS-PAGE analysis**.** The purity of the recombinantly expressed proteins was analyzed with SDS-PAGEs using 13% acrylamide gels (**Figure S8**).^6^ The samples were mixed with 6x loading buffer (375 mM TRIS-HCl pH 6.8, 9% SDS, 50% glycerol, 0.03% bromophenol blue and 9% (v/v) β-mercaptoethanol) and were incubated at 99 °C for 10 min. After that, the samples were run via a Mini-PROTEAN Tetra Cell System (Biorad, Vienna, Austria) on an SDS-PAGE gel. A molecular weight marker (Precision Plus Protein Dual Color Standard (Biorad)) was applied. Following Coomassie staining (0.02% Coomassie brilliant blue G-250, 5% aluminium sulfate, 10% ethanol, and 2 g/L *o*-phosphoric acid) overnight and the gels were washed in 10% ethanol and 20 g/L *o*-phosphoric acid.

1.6 Intact protein mass spectrometry**.** All purified proteins were analyzed using an UltiMate 3000 Nano LC-system coupled to an LTQ-Orbitrap Velos or Q Exactive mass spectrometer (Thermo Fisher Scientific, Bremen, Germany) equipped with a nanospray ion source (**Figure S9-S12**). All measurements were executed in positive mode. For calibration of the mass analyzer, ESI-L Low Concentration Tuning Mix, Part Number G1969-85000 (Agilent, Santa Clara, United States) was used. The enzyme solutions were rebuffered to 5 mM ammonium acetate at pH 7.4 by a Vivaspin 500 ultrafiltration device with a 30 kDa molecular weight cut-off (Sartorius). The enzymes were diluted to 1 µM in an aqueous solution containing 2% (v/v) acetonitrile and 1% (v/v) formic acid before being loaded on an LTQ Orbitrap Velos/Q-Exactive mass spectrometer (Thermo Fisher Scientiﬁc, Bremen, Germany) equipped with a nanospray ion source (ion transfer capillary temperature: 300 °C; electrospray voltage: 2.1 kV). The samples were injected into a trap column and separation was performed on a C4 analytical column (15 cm × 75 μm Accucore C4, 2.6 μm particle size, 150 Å pore size from Thermo Fisher) at a ﬂow rate of 300 nL/min. The mobile phase A comprised 2% acetonitrile, 98% H_2_O, and 0.1% formic acid. The mobile phase B comprised 80% acetonitrile, 20% H_2_O, and 0.1% formic acid.

1.7 Determination of extinction coefficients**.** Using a TECAN infinite M200 reader (Tecan, Salzburg, Austria), absorption curves were recorded at RT in a 96-well microplate. In a total volume of 200 µl, 2-amino-4-methylphenol (2A4MP) (**Figure**1, **(4)**) at concentrations of 0.05 mM, 0.10 mM, 0.2 mM, 0.50 mM and 1.00 mM was oxidized in TRIS-HCl buffer (50 mM TRIS, 200 mM NaCl, pH 7.5) containing 6.25 mM NaIO_4_. This oxidation produced the phenoxazinone product 3-amino-1,4a-dihydro-14a,8-dimethyl-2*H*-phenoxazin-2-one (APODM) (**Figure**1, **(6)**), whose extinction coefficients (6571 M⁻¹cm⁻¹ (R^2^=0.99) at 400 nm and 3621 M⁻¹cm⁻¹ (R^2^=0.99) at 340 nm) were subsequently determined spectrophotometrically in triplicates according to the Lambert-Beer law^5^ by calculating a linear regression.

For determining the extinction coefficient of the nitroso reaction product 4-methyl-2-nitrosophenol (4M2NP, **Figure**1**, (8)**), 2A4MP (**Figure 1**, **(4)**, 0.05 mM, 0.10 mM, 0.2 mM, 0.50 mM, 1.00 mM) was enzymatically oxidized in TRIS-HCl buffer by adding 50 µg/ml *Sg*GriF in a total volume of 200 µl, which yielded a mixture of APODM (**Figure 1**, **(6)**) and 4M2NP (**Figure 1, (8)**). To quantify the amount of 2A4MP (**Figure 1**, **(4)**) converted into APODM (**Figure 1**, **(6)**), 6.25 mM NaIO_4_ was added after the enzymatic oxidation process has been completed which oxidized any residual 2A4MP (**Figure 1**, **(4)**) into APODM (**Figure 1**, **(6)**). By measuring absorbance at 400 nm, the amount of 2A4MP (**Figure 1**, **(4)**) converted into APODM (**Figure 1**, **(6)**) was calculated using the Lambert-Beer law and the extinction coefficient of APODM (**Figure 1**, **(6)**) at 400 nm (6571 M^-1^cm^-1^).^5^ This allowed determining the APODM (**Figure 1**, **(6)**) amount present in the reaction mixture. Furthermore, the total absorbance at 340 nm has been measured, which represents the cumulative absorbance of APODM (**Figure 1**, **(6)**) and 4M2NP (**Figure 1, (8)**). To extract the partial absorbance of 4M2NP (**Figure 1, (8)**) at 340 nm, the partial absorbance of APODM (**Figure 1**, **(6)**) corresponding to its previously determined concentration has been calculated using its extinction coefficient at 340 nm (3621 M^-1^cm^-1^) and subtracted from the total absorbance at 340 nm (**Figure SA**). Using this method, the extinction coefficient of 4M2NP (**Figure 1, (8)**) at 340 nm (6428 M^-1^cm^-1^) was determined according to Lambert-Beer law.^5^


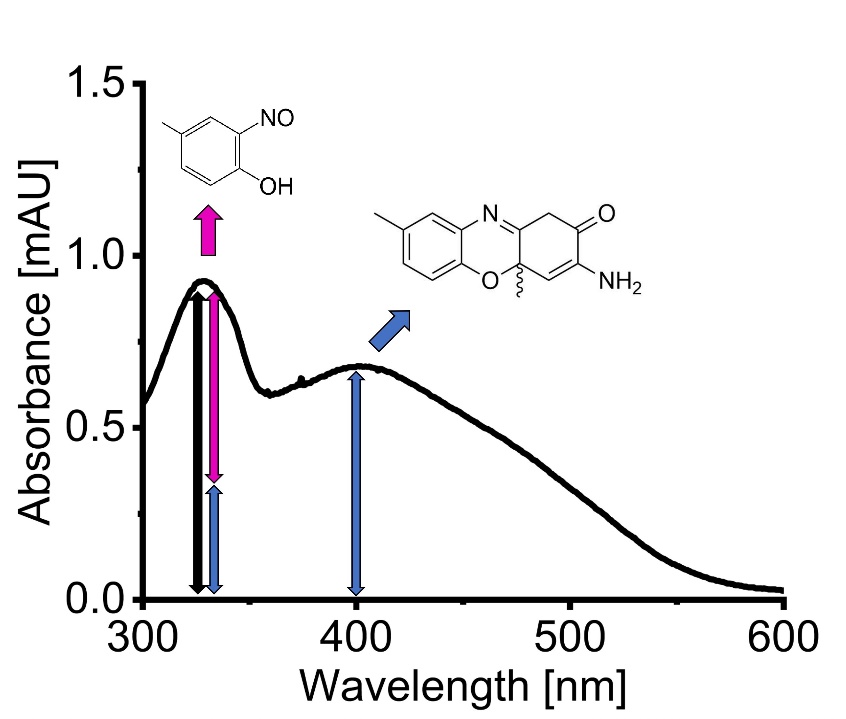


**Figure SA:** Quinone-imine forming activity (blue bar) vs. nitrosophenol forming activity (magenta bar). Cumulative absorbance of APODM (**Figure 1**, **(6)**) and 4M2NP (**Figure 1, (8)**) depicted in black bar.

1.8 Substrate activity assays**.** By detecting the appearance of APODM (**Figure 1**, **(6)**, **A**: quinone imine-forming activity) at 400 nm and 4M2NP (**Figure 1**, **(8)**, **B**: nitroso-forming activity) at 340 nm, which is produced by the enzymatic oxidation of 2A4MP (**Figure 1**, **(4)**) in the presence of molecular oxygen, the specific activities of recombinantly expressed proteins were determined spectrophotometrically. Enzymatic activities were assayed at RT using a TECAN infinite M200 reader (Tecan, Salzburg, Austria) in a 96-well microplate. Each reaction had a total volume of 200 µl and contained 10 mM 2A4MP (**Figure 1**, **(4)**), variable amounts of enzyme (5 µg/ml for *Sg*GriF or *Sg*GriF-Y47V; 50 µg/ml for *Sg*GriF*-*N43I, *Sz*TYR or *Sz*TYR-I42N) and TRIS-HCl buffer (50 mM TRIS, 200 mM NaCl, pH 7.5). All kinetic measurements were performed in triplicates. Quinone imine-forming activities (**Figure 1: A**) were determined directly by measuring the slopes of the linear portion of the absorbance-time curve at 400 nm.^7,8,9^ Nitroso-forming activities (**Figure 1: B**) were determined by measuring absorbance-time curve at 400 nm and at 340 nm, as both quinone imine reaction products and nitroso reaction products are formed simultaneously. Absorbance-time curves recorded at 400 nm allowed to monitor the formation of APODM (**Figure 1**, **(6)**). The corresponding partial absorbance of APODM (**Figure 1**, **(6)**) at 340 nm could be calculated using the Lambert-Beer law and its extinction coefficient at 340 nm (3621 M^-1^cm^-1^).^5^ Thus, the formation of 4M2NP (**Figure 1, (8)**) could be monitored by subtracting partial absorbance values (at 340 nm) of APODM (**Figure 1**, **(6)**) from the total absorbance values at 340 nm (**Figure SA**). The partial absorbance values of 4M2NP (**Figure 1, (8)**) and its extinction coefficient at 340 nm (6428 M^-1^cm^-1^) were used to calculate nitroso-forming activity according to the Lambert-Beer law.^5^

1.9 Evolution of oxidized 2A4MP (**Figure 1**, **(1)**)**.** Absorption spectra between 200 nm and 800 nm were recorded on a Shimadzu UV-1800 spectrophotometer (Shimadzu, Kyoto, Japan) using a 600 μl quartz cuvette at 25 °C which were kept at 25 °C by a Julabo F25 MH thermostat in a circulating water-bath (JULABO GmbH, Seelbach, Deutschland). A full absorption spectrum ranging from 200-800 nm was reached within a timescale of 111 s (**Figure S14**). A total volume of 200 µl was used for the time resolved absorption spectra, containing TRIS-HCl buffer (50 mM TRIS, 200 mM NaCl, pH 7.5), 50 µg/ml enzyme (*Sg*GriF, *Sg*GriF-N43I, *Sg*GriF-Y47V, *Sz*TYR or *Sz*TYR-I42N) and 200 µM 2A4MP (**Figure 1**, **(4)**). Time resolved absorption curves (evolution of oxidized 2A4MP (**Figure 1**, **(4)**) of *Sg*GriF contained TRIS-HCl buffer (50 mM TRIS, 200 mM NaCl, pH 7.5), 5 µg/ml enzyme (*Sg*GriF, *Sg*GriF-N43I, *Sg*GriF-Y47V, *Sz*TYR or *Sz*TYR-I42N) and 300 µM 2A4MP (**Figure 1, (4)**).

1.10 Docking Experiments. Molecular docking was performed using AutoDock Vina (https://vina.scripps.edu/). The structure of the substrates 2A4MP was obtained from the PDB as a .pdb file and formatted into .pdbqt files using AutoDockTools (ADT, v. 1.5.6, https://autodocksuite.scripps.edu/adt/). The structure files (.pdb format) of *Sz*TYR and *Sg*GriF were obtained from the AlphaFold DB (*Sg*GriF: AlphaFoldDB model B1VTI5, *Sz*TYR: AlphaFoldDB model A0A429IXZ5). Binding poses were searched in a grid box of 15 × 15 × 15 Å^3^ (spacing = 1.0 Å) centered in-between the two copper ions with the exhaustiveness set to 100.

# Results

## Supplementary Figures


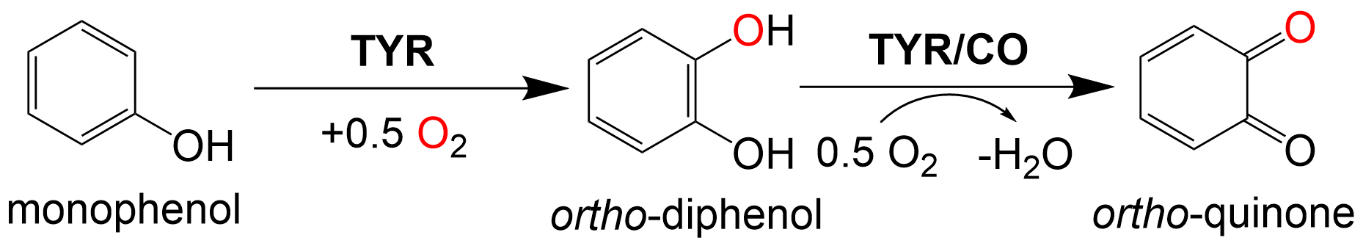


Figure S1 Monophenolase and diphenolase activity**:** TYR catalyzes the oxidation of monophenol to *o*-diphenol (monophenolase activity, EC 1.14.18.1) and thereafter the oxidation to *o*-quinone (diphenolase activity, EC 1.10.3.1). Catechol oxidases (CO) only performs diphenenolase activity with no monophenolase activity.


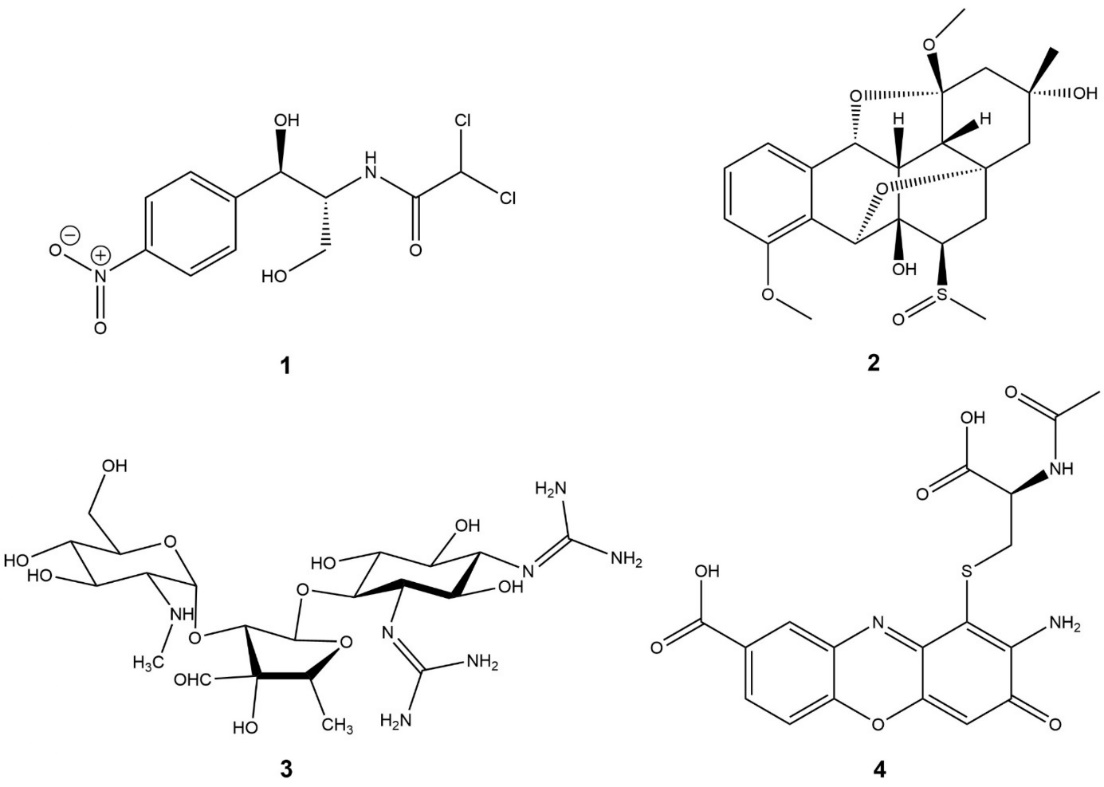


Figure S2 Bioactive substances**:** Natural compounds produced by *Streptomyces venezuelae*: Chloramphenicol^10^ (**1**) and by *Streptomyces griseus*: Grisemycin^11^ (**2**), Streptomycin^12^ (**3**) and Grixazone B^8^ (**4**).

**
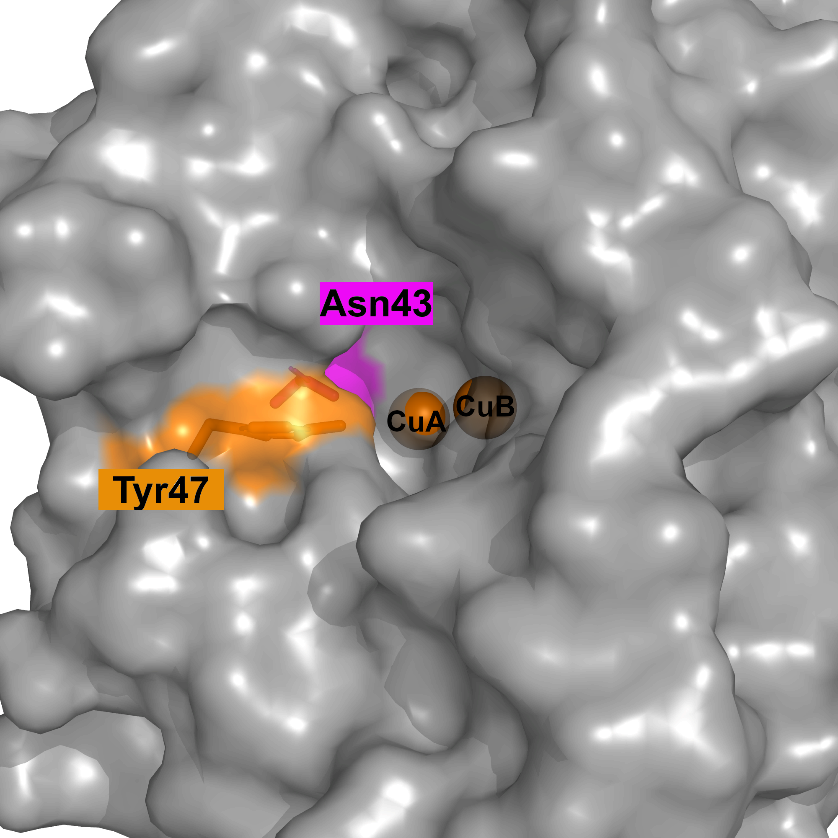
**

Figure S3 Binding pocket of SgGriF**:** AlphaFoldDB^13,14^ model (B1VTI5) of *Sg*GriF shows surface area at the catalytic center.^10,11^ The two copper ions are depicted as spheres in orange. The highly conserved residue Asn43 is highlighted in magenta and highly conserved Tyr47 is highlighted in orange on the surface area. Depicted model is taken from AlphaFold DB^13,14^ and visualized with PyMOL Molecular Graphics System, Version 3.0 Schrödinger, LLC.

**
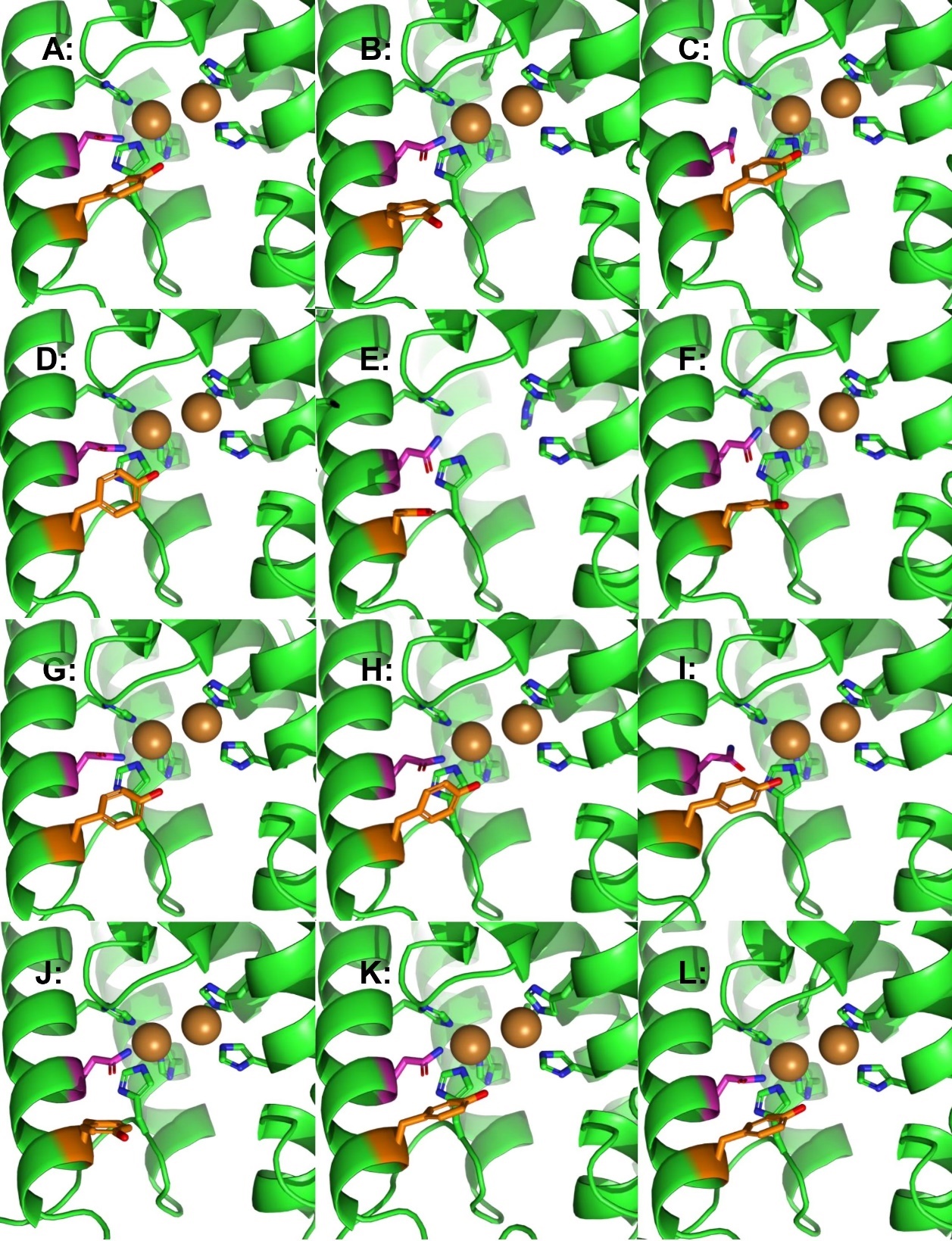
**

Figure S4 Structural alignments of AOs**:** Catalytic center of **A**: *Sg*GriF (UniprotKB: B1VTI5), **B**: *Sl*fevF (UniprotKB: A0A2H5BVB1), **C**: *Sm*AO (UniprotKB: A0A1W7D1S4), **D**: *Sm*NspF (UniprotKB: D6RTB9), **E**: *Ss*AO (UniprotKB: A0A0W7WYA7), **F**: *Sc*AO (UniprotKB: A0A1Q4VUB3), **G**: *Se*AO (UniprotKB: A0A197SSE1), **H**: *Smp*AO (UniprotKB: A0A1V2RQ55), **I**: *Sr*AO (UniprotKB: A0A2S4YIE3), **J**: *St*bagH (UniprotKB: A0A482LS25), **K**: *Swac*AO (UniprotKB: A0A429IXZ5), **L**: *Sw*fevF (UniprotKB: L0N6P7).^10,11^ Asn residues are depicted in magenta and Tyr residues are depicted in orange. All nitrogen atoms are depicted in blue and oxygens in red. All depicted models are taken from AlphaFold DB^13,14^ and visualized with PyMOL Molecular Graphics System, Version 3.0 Schrödinger, LLC.


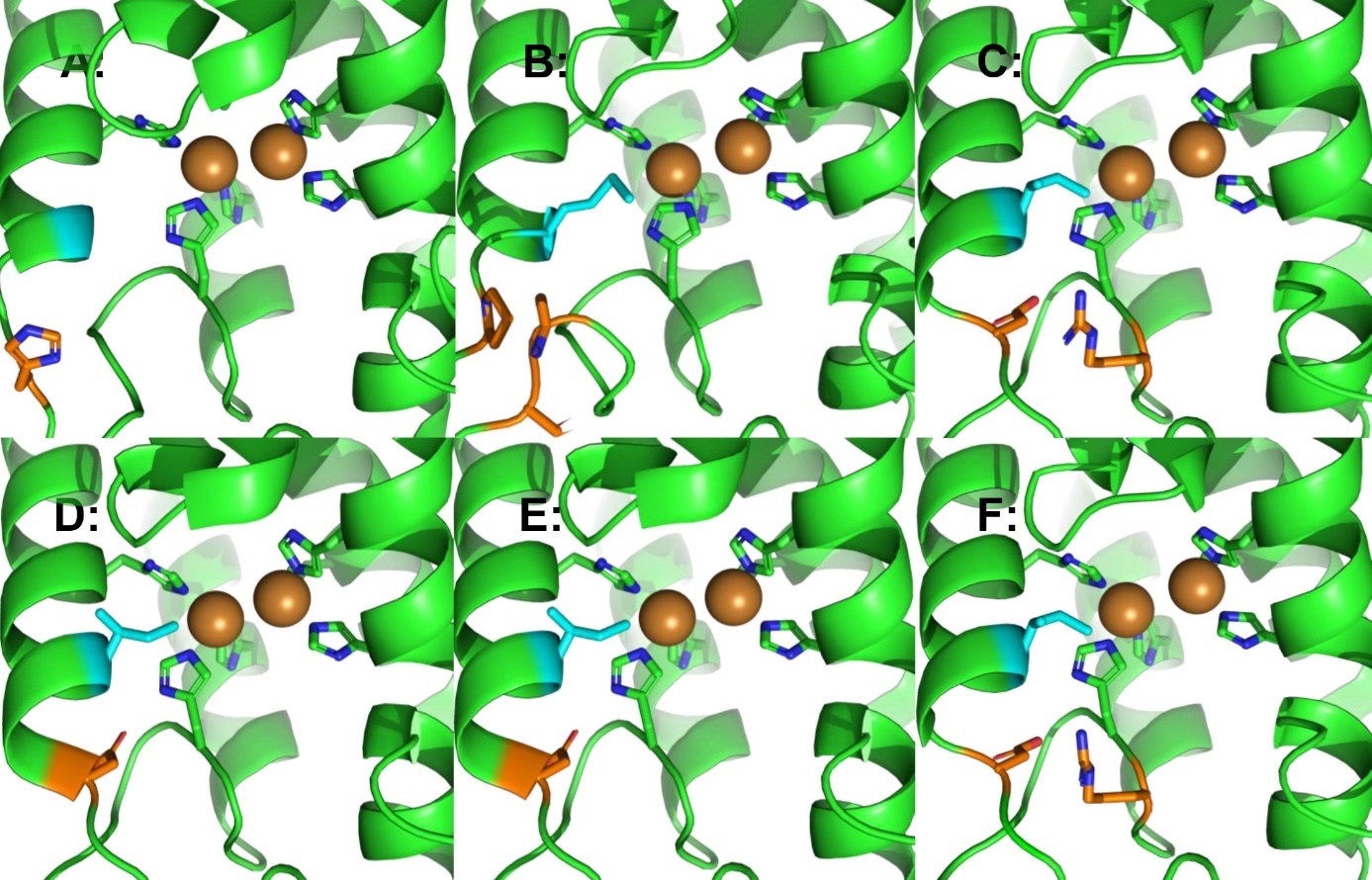


Figure S5 Structural alignments of TYRs**:** Catalytic center of **A**: *Bm*TYR (PDB: 3NM8)^15^, **B**: *SinA*TYR (UniprotKB: L0D705)^13,14^, **C**: *Sa*TYR (PDB: 6J2U)^16^, **D**: *Sc*TYR (PDB: 1WX2)^13,14^, **E**: *Sk*TYR (UniprotKB: A0A077HD11)^13,14^, **F**: *Sz*TYR^13,14^ (UniprotKB: A0A2S3Y8X7). Homologous residues to Asn43 of *Sg*GriF are depicted in cyan and residues in the vicinity (up to 2 Å) to Tyr47 of *Sg*GriF are highlighted in orange. All nitrogen atoms are depicted in blue and oxygens in red. Depicted crystal structures are taken from RCSB Protein Data Bank (PDB) and models from AlphaFold DB^13,14^ and visualized with PyMOL Molecular Graphics System, Version 3.0 Schrödinger, LLC.

***
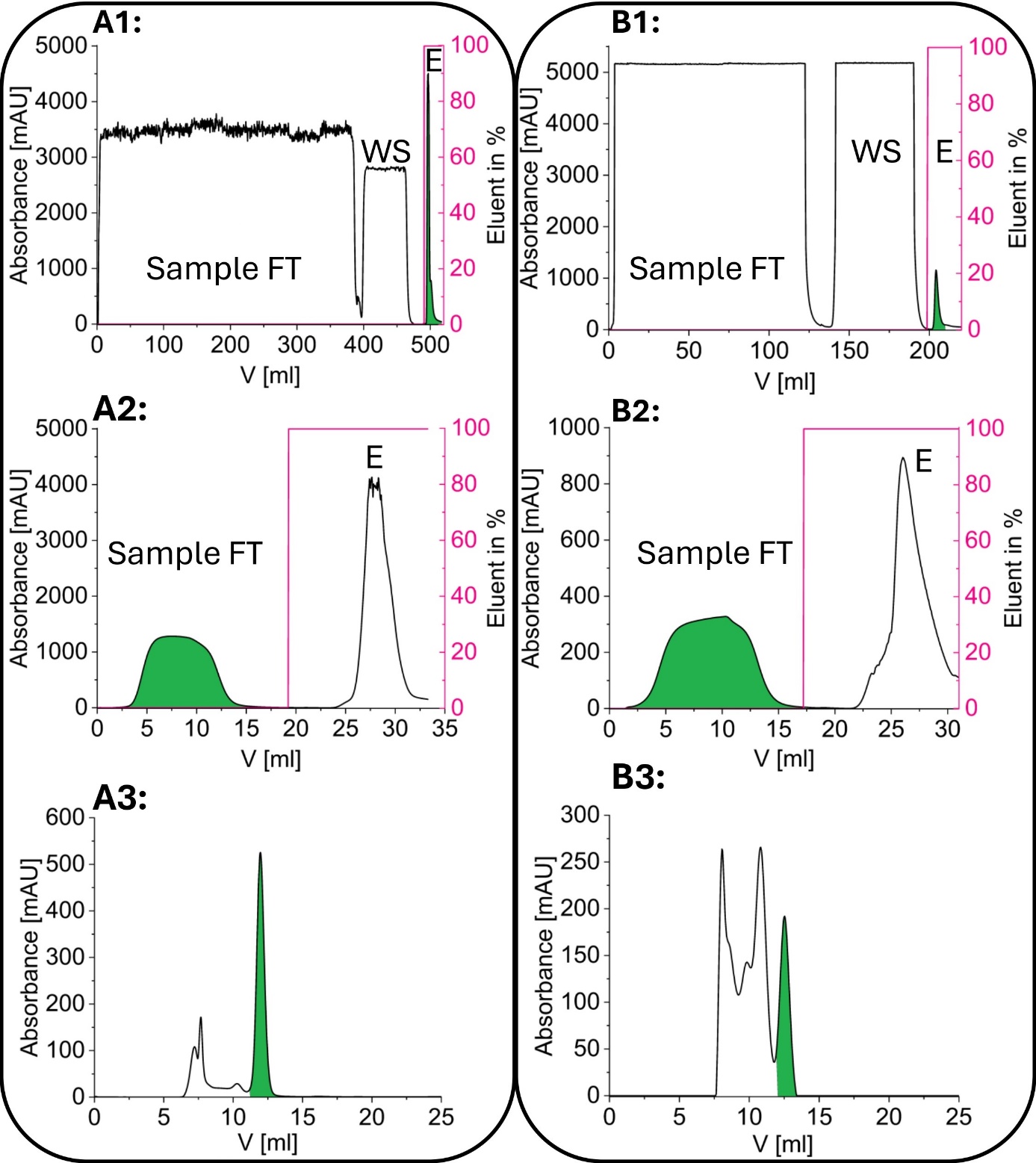
***

Figure S6 Purification of SgGriF and SgGriF-Y47V**: A1**: First affinity chromatography with GSTrap FF (GE) of *Sg*GriF GST-Tag fusion protein, **A2**: Second affinity chromatography after GST-cleavage with GSTrap FF (GE) of *Sg*GriF (FT (Flow Through), WS (Washing Step), E (Elution Step)), **A3**: Size exclusion chromatography of *Sg*GriF, **B1**: First affinity chromatography of *Sg*GriF-Y47V GST-Tag fusion protein, **B2**: Second affinity chromatography of *Sg*GriF-Y47V, and **B3**: Size exclusion chromatography of *Sg*GriF-Y47V. Collected fractions of targeted proteins are colored in green. Experimental procedures are under section 1.3.

**
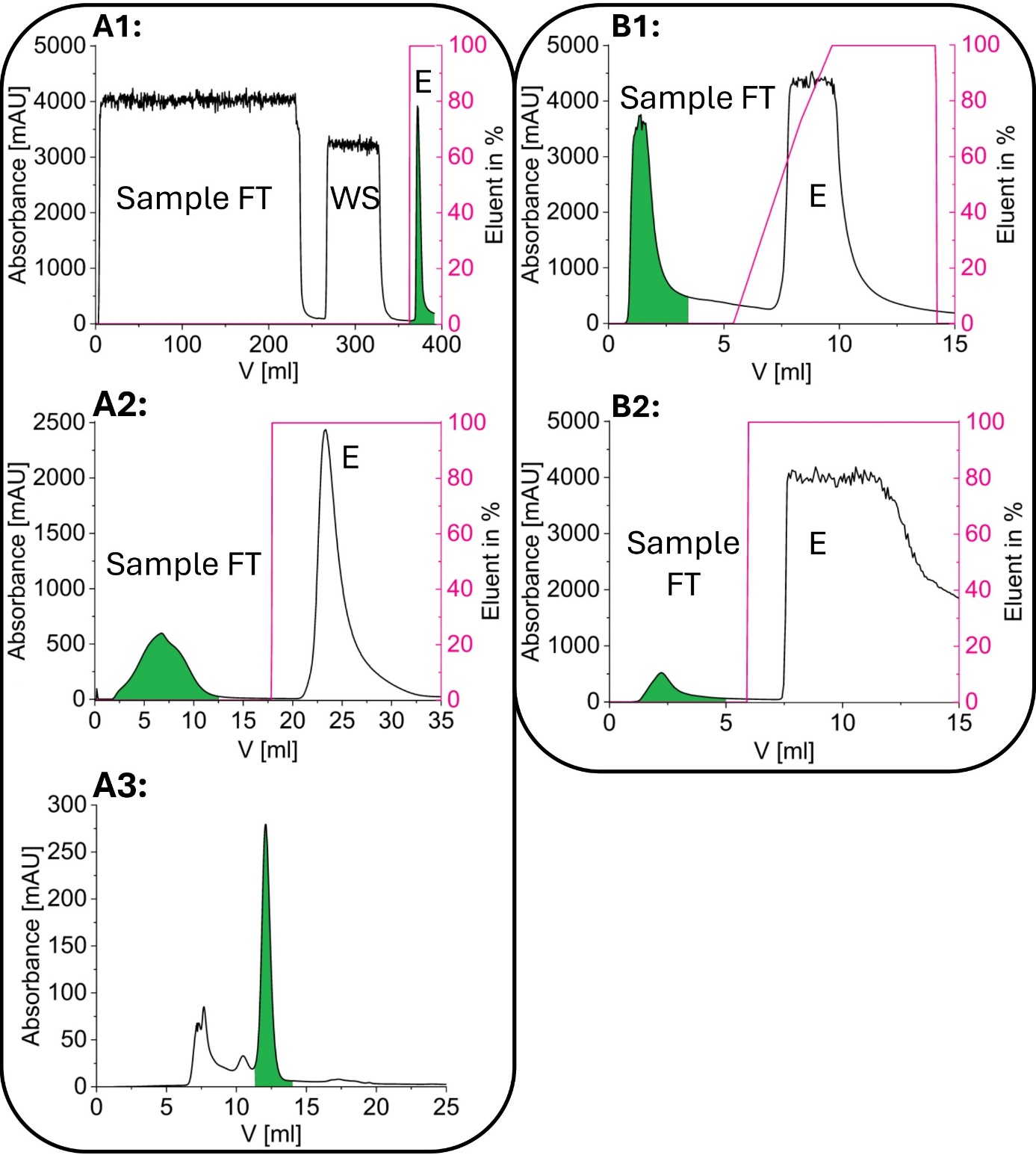
**

Figure S7 Purification of SgGriF-N43I, SzTYR and SzTYR-I42N**: A1**: First affinity chromatography with GSTrap FF (GE) of *Sg*GriF-N43I GST-Tag fusion protein, **A2**: Second affinity chromatography GST-cleavage with GSTrap FF (GE) of *Sg*GriF-N43I (FT (Flow Through), WS (Washing Step), E (Elution Step)), **A3**: Size exclusion chromatography of *Sg*GriF-N43I. **B1**: Anion exchange chromatography of *Sz*TYR with gradient from 0 to 100% elution buffer between minute 5.5-9.5. **B2**: Anion exchange chromatography of *Sz*TYR-I42N. Collected fractions of targeted proteins are colored in green. Experimental procedures are under section 1.3 and 1.4.


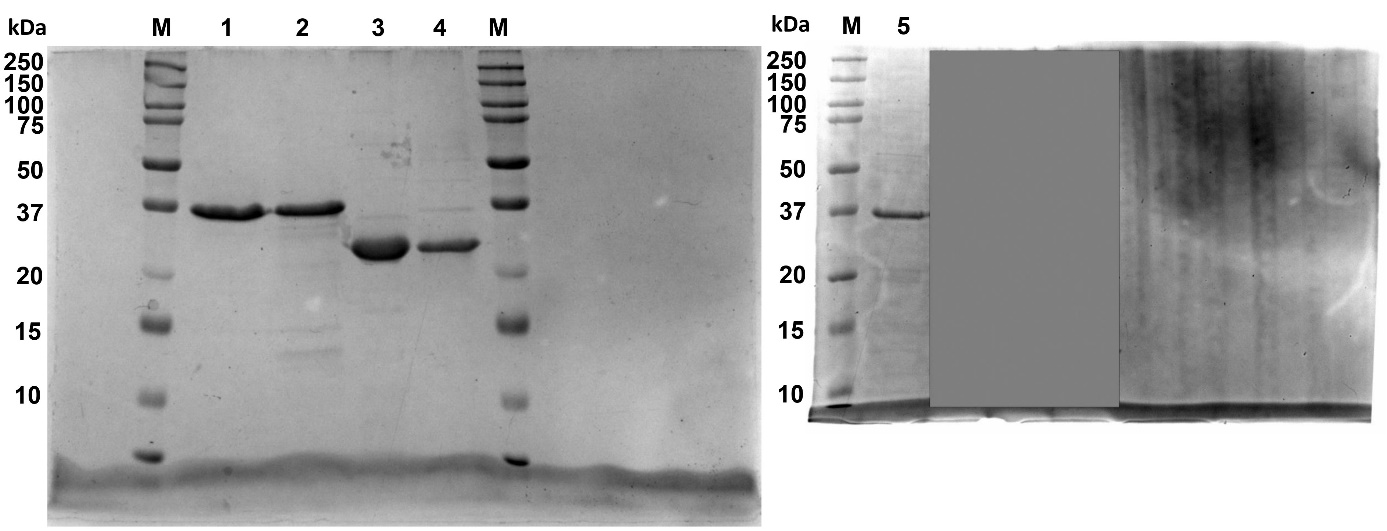


Figure S8 Reducing SDS-PAGE (13 %, untruncated) of recombinant proteins**:** **1**: *Sg*GriF, **2**: *Sg*GriF-N43I, **3**: *Sz*TYR, **4**: *Sz*TYR-I42N and **5**: *Sg*GriF-Y47V. The lanes indicated with **M** contain the molecular weight marker (Precision Plus Protein Dual Color Standards (Bio-Rad Laboratories GmbH, Feldkirchen, Germany)); the size of the standard bands is given in kDa.


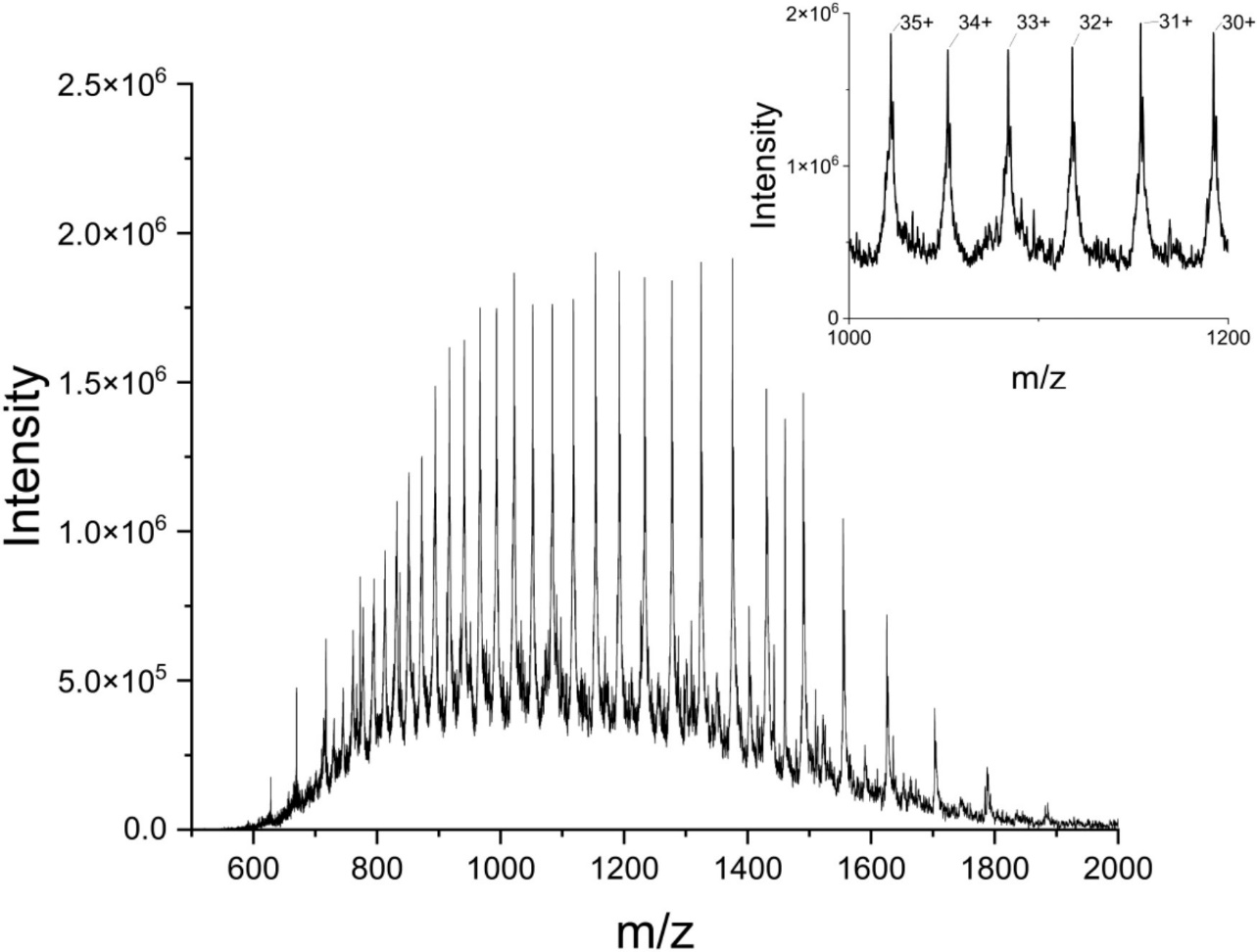


Figure S9 ESI-MS of SgGriF**:** ESI-LTQ-Orbitrap Velos mass spectrum of *Sg*GriF in positive mode. Theoretical mass: 35737.28 Da, experimental mass: 35736.51 ± 0.96 Da. Signal-to-Noise Ratio (S/N): 21, Normalization Level (NL): 1.93E + 006. To the left the entire mass spectrum of *Sg*GriF is depicted, on the upper right a zoom-in to high intense signals originating from the pseudomolecular ions [M + 35H]^35+^ to [M + 30H]^30+^ is shown.

**
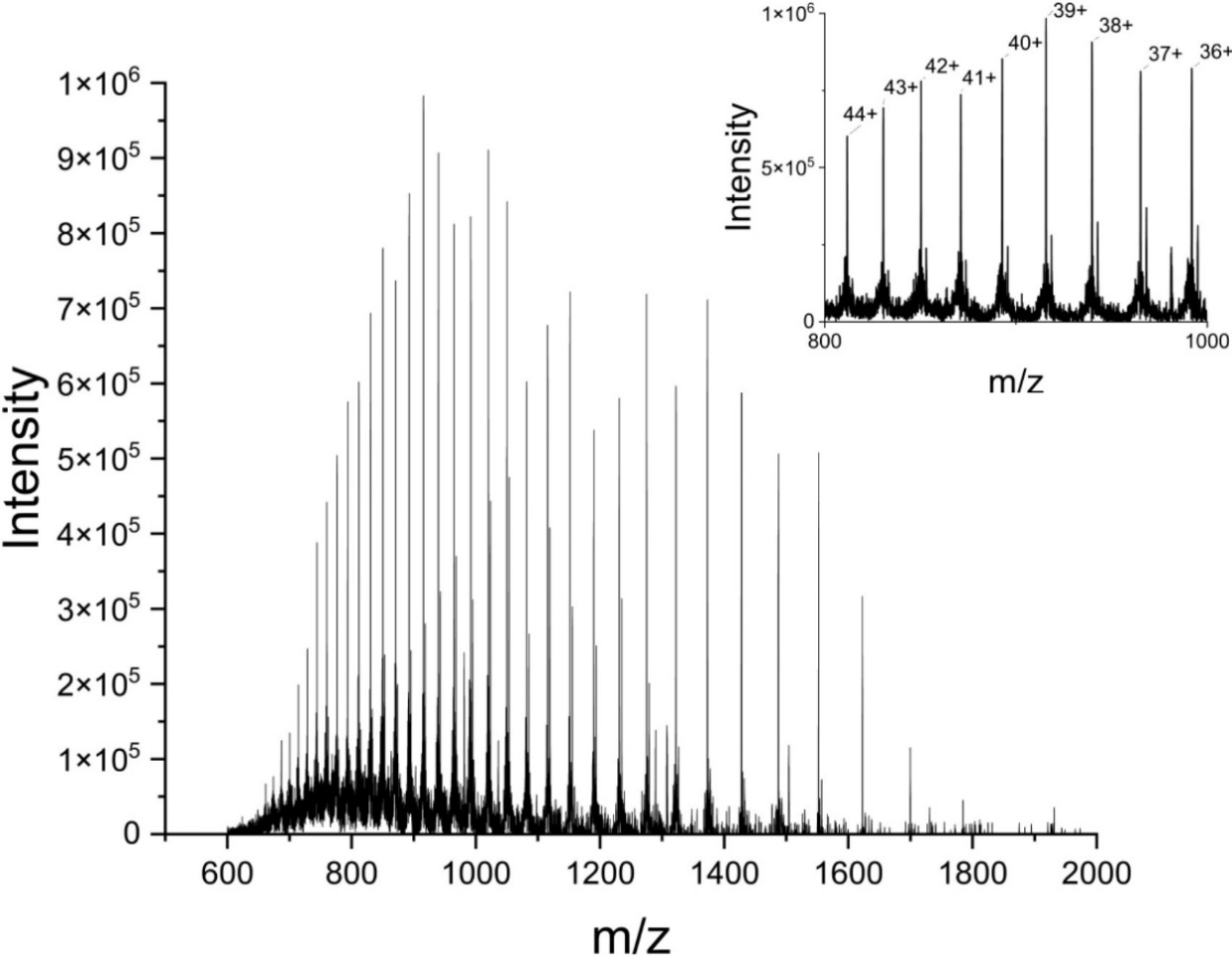
**

Figure S10 ESI-MS of SgGriF-Y47V**:** ESI-Q-Exactive mass spectrum of *Sg*GriF-Y47V in positive mode. Theoretical Mass: 35673.17 Da, experimental mass: 35672.17 ± 1.37 Da. Signal-to-Noise Ratio (S/N): 61, NL: 9.83E + 005. To the left the entire mass spectrum of *Sg*GriF-Y47V is depicted, on the upper right a zoom-in to the most intense signals originating from the pseudomolecular ions [M + 44H]^44+^ to [M + 36H]^36+^ is shown.

**
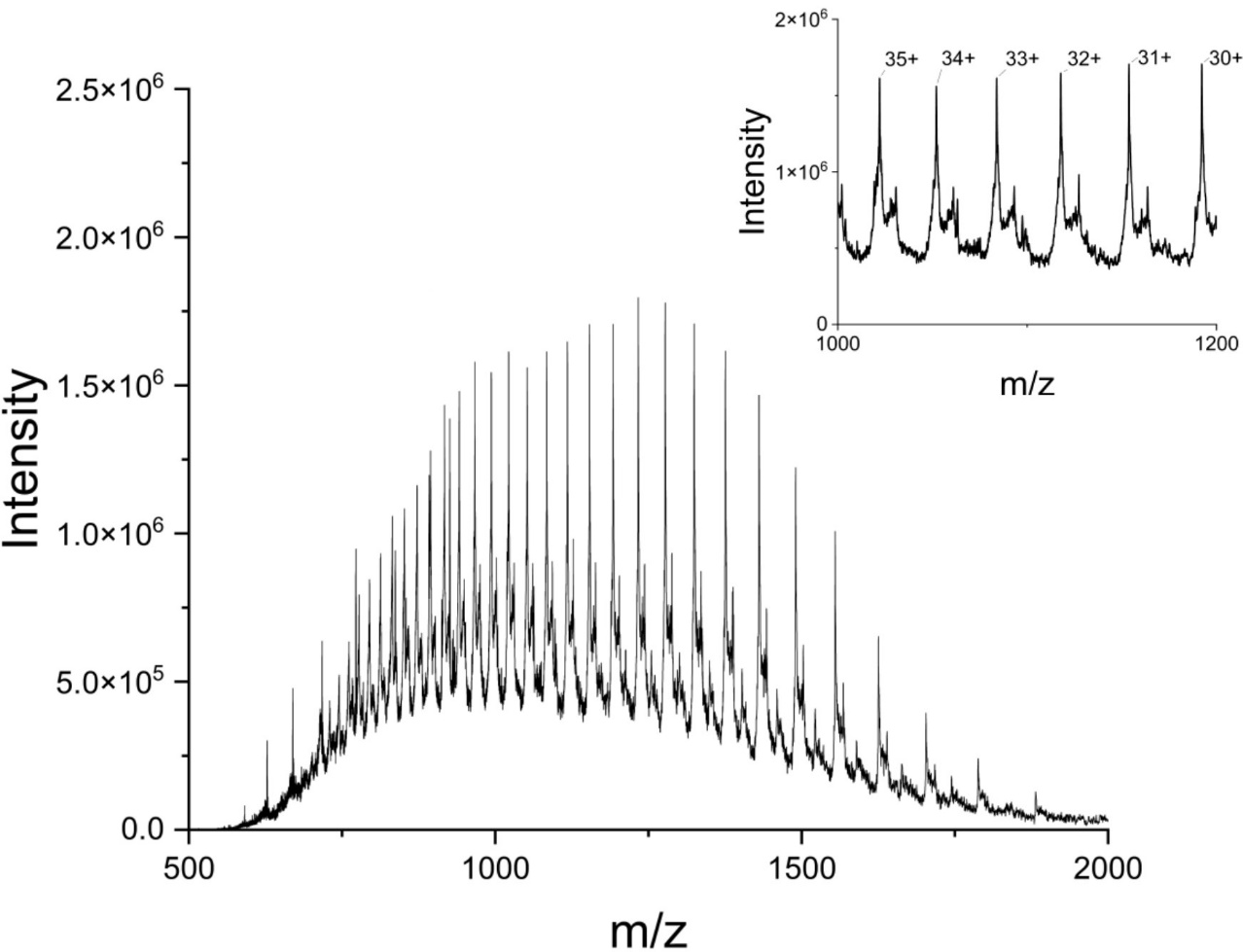
**

Figure S11 ESI-MS of SgGriF-N43I**:** ESI-LTQ-Orbitrap Velos mass spectrum of *Sg*GriF-N43I in positive mode. Theoretical Mass: 35736.36 Da, experimental mass: 35735.64 ± 1.03 Da. Signal-to-Noise Ratio (S/N): 12, NL: 1.80E + 006. To the left the entire mass spectrum of *Sg*GriF-N43I is depicted, on the upper right a zoom-in to high intense signals originating from the pseudomolecular ions [M + 35H]^35+^ to [M + 30H]^30+^ is shown.

**
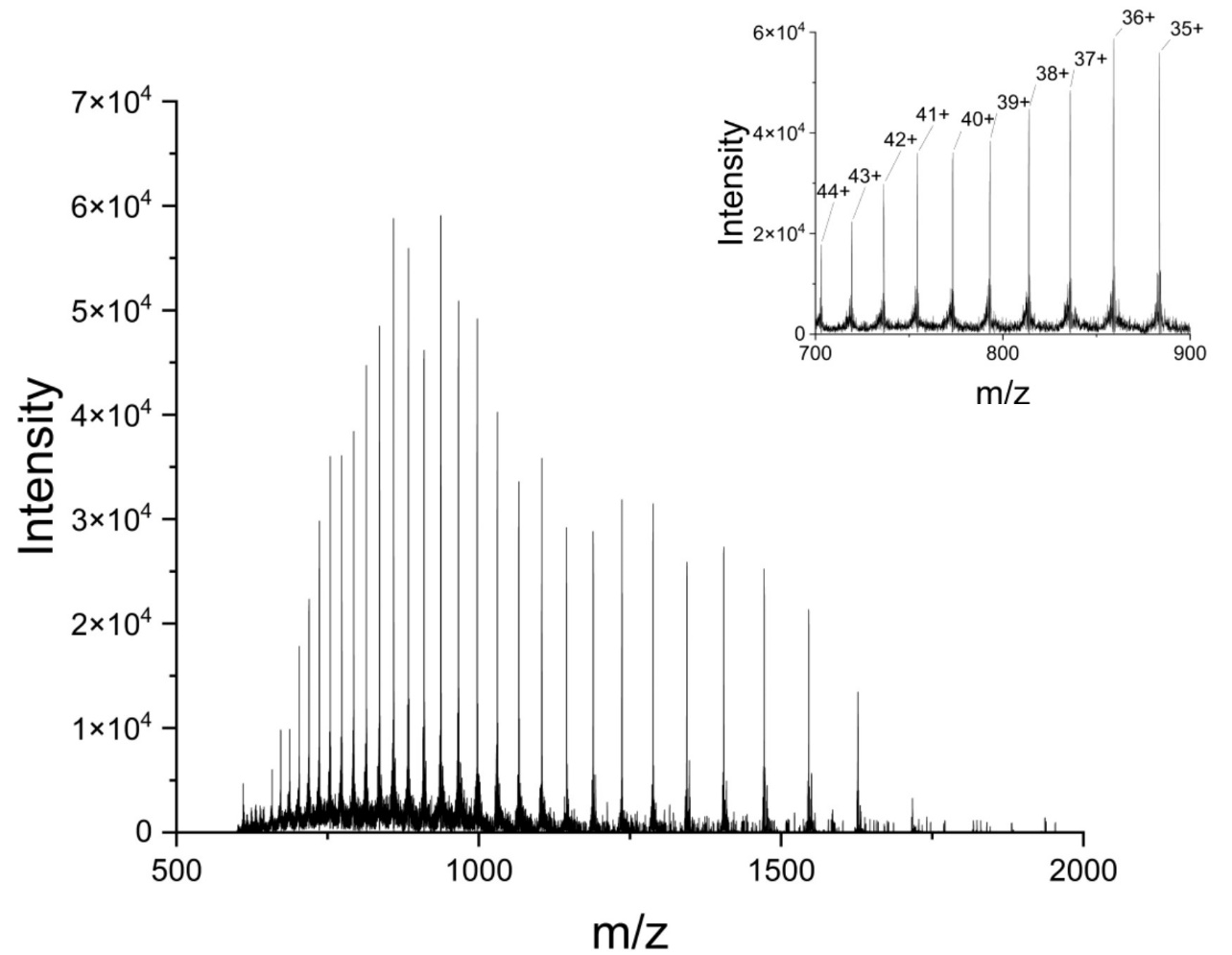
**

Figure S12 ESI-MS of SzTYR**:** ESI-Q-Exactive mass spectrum of *Sz*TYR in positive mode. Theoretical Mass: 31032.79 Da, theoretical Mass (methionine cleavage by methionine aminopeptidase): 30892.59 Da, experimental mass: 30892.60 ± 1.20 Da. Signal-to-Noise Ratio (S/N): 356, NL: 3.11E+006.^17^ To the left the entire mass spectrum of *Sz*TYR is depicted, on the upper right a zoom-in to the most intense signals originating from the pseudomolecular ions [M + 44H]^44+^ to [M + 35H]^35+^ is shown.

**
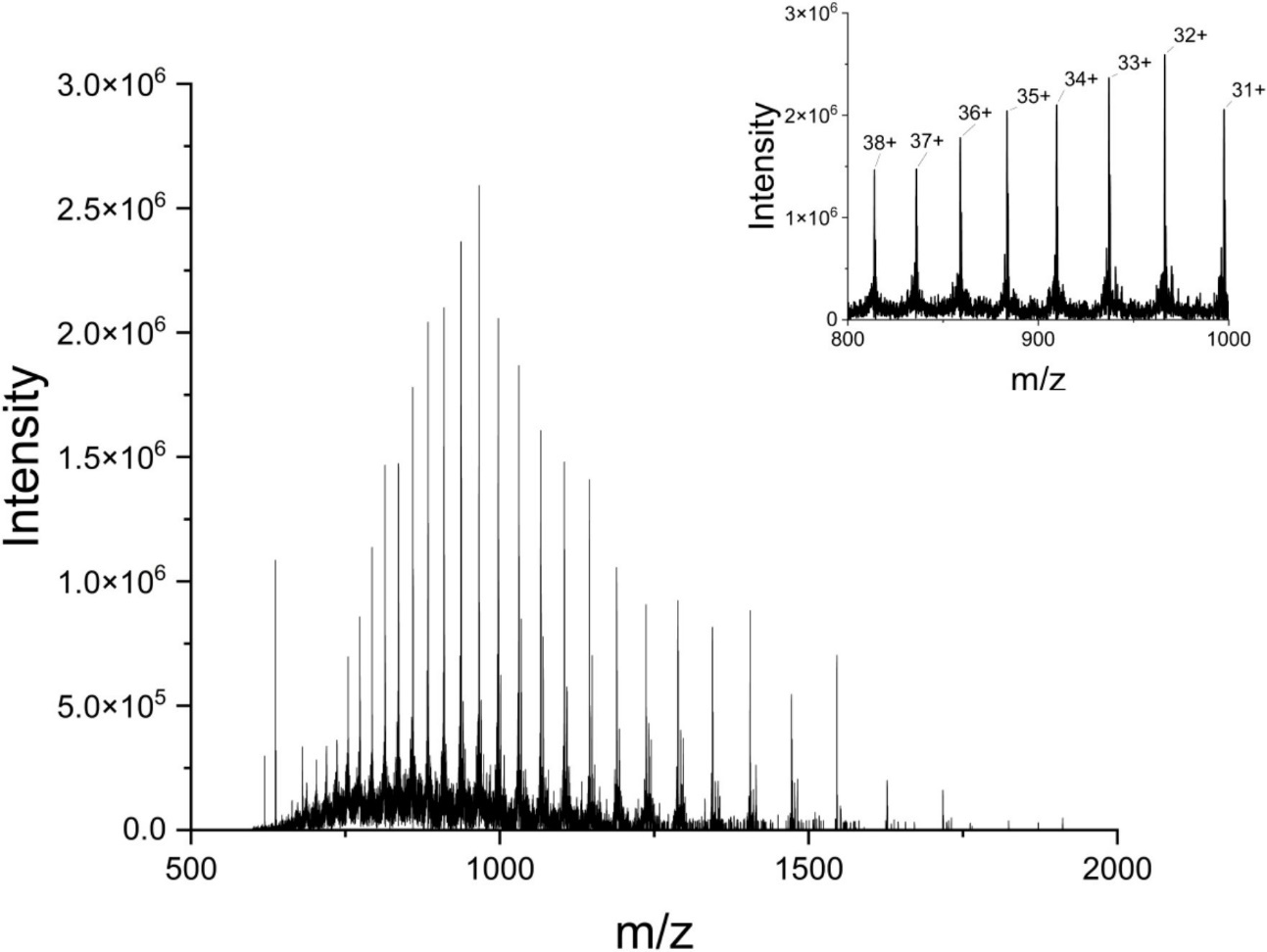
**

Figure S13 ESI-MS of SzTYR-I42N**:** ESI-Q-Exactive mass spectrum of *Sz*TYR-I42N in positive mode. Theoretical Mass: 31024.73 Da, theoretical Mass (methionine cleavage by methionine aminopeptidase): 30893.53 Da, experimental mass: 30893.48 ± 0.99 Da. Signal-to-Noise Ratio (S/N): 173, NL: 2.59E+006.^17^ To the left the entire mass spectrum of *Sz*TYR-I42N is depicted, on the upper right a zoom-in to the most intense signals originating from the pseudomolecular ions [M + 38H]^38+^ to [M + 31H]^31+^ is shown.


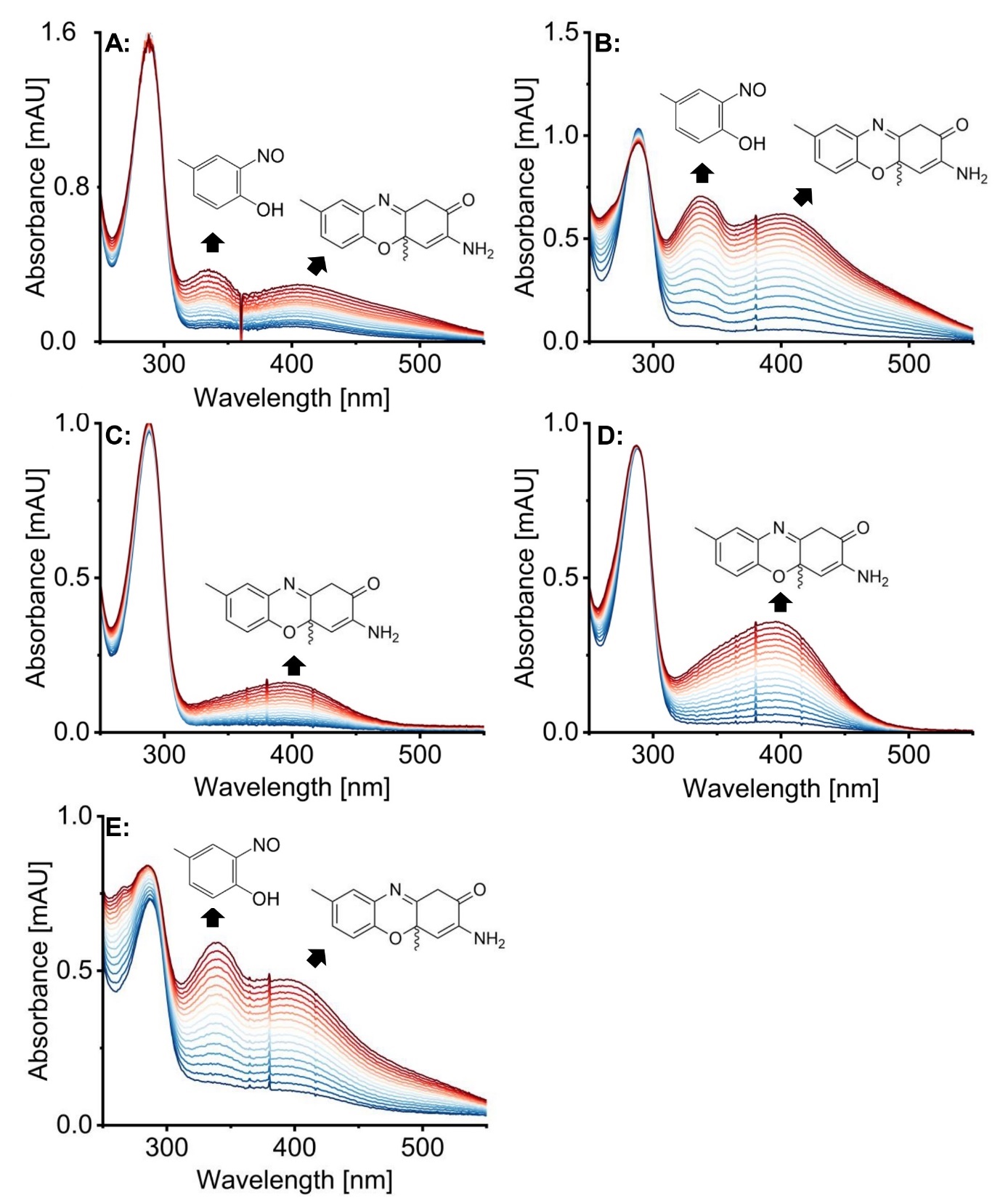


Figure S14 Evolution of oxidized 2A4MP absorption spectra in TRIS-HCl buffer**:** Absorption spectra recorded between 250 nm and 550 nm of 2A4MP (200 µM/300 µM) oxidized with **A**: 5 µg/ml *Sg*GriF, **B**: 50 µg/ml *Sg*GriF-Y47V, **C**: 50 µg/ml *Sg*GriF-N43I **D**: 50 µg/ml *Sz*TYR, **E**: 50 µg/ml *Sz*TYR-I42N in a quartz cuvette. Each spectrum is recorded with a time interval of 111 s starting from first spectra in dark blue to 16^th^ spectra in dark red. The light source change of the Shimadzu UV-1800 spectrophotometer (Shimadzu) in **A** was at 360 nm and in **B**, **C**, **D** and **E** at 380 nm.

# Supplementary Tables

## Table S1 Designed primers with annealing temperatures.

| **Name** | **Sequence** | **Annealing T** |
| --- | --- | --- |
| *Sg*GriF-FWD | 5΄ agctcgtctccaATGGTCCACGTACGCAAGAAC 3΄ | 68 °C |
| *Sg*GriF-REV | 5΄ agctcgtctcatcccCTACTGGTCGTAGGTGTAGAACCG 3΄ | 68 °C |
| *Sg*GriF-Y47V-FWD | 5΄ ACGGAC**GT**CCTCGACAAGG 3΄ | 67 °C |
| *Sg*GriF-Y47V-REV | 5΄ GGAGTTGATCTGTATGTGGAGCTTG 3΄ | 67 °C |
| *Sg*GriF-N43I-FWD | 5΄ ACGGACTACCTCGACAAGGAG 3΄ | 68 °C |
| *Sg*GriF-N43I-REV | 5΄ GGAG**A**TGATCTGTATGTGGAGCTTG 3΄ | 67 °C |
| *Sz*TYR-I42N-FWD | 5΄ CAACGCCTTCA**A**CATGAGCGATAC 3΄ | 68 °C |
| *Sz*TYR-I42N-REV | 5΄ TGCGTGGTGACGAACGAG 3΄ | 68 °C |

Note: Non-pairing bases are written in small letters and mutated bases are in bold letters.

## Table S2 Purification of expressed enzymes.

| **Enzyme** | **Purification Step** | **Activity* [Umg^-1^]** | **Total Activity [U]** | **Yield [%]** |
| --- | --- | --- | --- | --- |
| ***Sg*GriF** | Crude Lysate | 0.0064 ± 0.0003 | 101 | 100 |
|  | GST-tagged Enzyme after AC | 1.4702 ± 0.1302 | 38 | 38 |
|  | GST-cleaved Enzyme after AC | 5.2140 ± 0.4581 | 13 | 13 |
|  | Purified Enzyme after SEC | 7.8138 ± 0.1705 | 6 | 6 |
| ***Sg*GriF-Y47V** | Crude Lysate | 0.0030 ± 0.0001 | 47 | 100 |
|  | GST-tagged Enzyme after AC | 0.4321 ± 0.0058 | 38 | 81 |
|  | GST-cleaved Enzyme after AC | 1.7335 ± 0.6052 | 26 | 55 |
|  | Purified Enzyme after SEC | 2.9664 ± 0.0303 | 5 | 10 |
| ***Sg*GriF-N43I** | Crude Lysate | 0.0009 ± 0.0002 | 14 | 100 |
|  | GST-tagged Enzyme after AC | 0.0243 ± 0.0038 | 10 | 72 |
|  | GST-cleaved Enzyme after AC | 0.3369 ± 0.0077 | 7 | 49 |
|  | Purified Enzyme after SEC | 0.5519 ± 0.0210 | 2 | 15 |
| ***Sz*TYR** | Extracellular Medium | 0.0016 ± 0.0001 | 15 | 100 |
|  | Purified Enzyme after AEX | 0.5990 ± 0.1409 | 10 | 69 |
| SzTYR-I42N | Extracellular Medium | **n.d. | - | - |
|  | Purified Enzyme after AEX | 0.0901 ± 0.0125 | - | - |

*Specific activities were determined by oxidation of 10 mM 2A4MP with 5 µg/ml of enzyme at 400 nm and the corresponding first standard deviations are given after the ± sign. **n.d.: Specific activities not determined due to low activites.

# Data Management Plan

https://phaidra.univie.ac.at/detail/o:2119942

# References

[1] F. Panis, R. F. Krachler, R., R. Krachler, A. Rompel, *Environ. Sci. Technol.* **2021**, *55*, 11445–11454. doi: 10.1021/acs.est.1c02514

[2] M. Pretzler, A. Bijelic, A. Rompel, *Sci. Rep.* **2017**, *7*, 1810. doi: 10.1038/s41598-017-01813-1

[3] K. R. Fourie, H. L. Wilson, *Vaccines* **2020**, *8*, 773. doi: 10.3390/vaccines8040773

[4] E. Gasteiger, C. Hoogland, A. Gattiker, S. Duvaud, M. Wilkins, R. Appel, A. Bairoch, in *Proteomics Protocols Handbook* (Ed.: J. Walker), Humana Press, Totowa **2005**, 571–607. doi: 10.1385/1-59259-890-0:571

[5] D. F. Swinehart, *J. Chem. Educ.* **1962**, *39*, 333. doi: 10.1021/ed039p333

[6] U. K. Laemmli, *Nature* **1970**, *227*, 680–685. doi: 10.1038/227680a0

[7] A. Tomoda, M. Arisawa, S. Koshimura, *J. Biochem.* **1991**, *110*, 1004-1007. doi: 10.1093/oxfordjournals.jbchem.a123669

[8] H. Suzuki, Y. Furusho, T. Higashi, Y. Ohnishi, S. Horinouchi, *J. Biol. Chem.* **2006**, *281*, 824–833. doi: 10.1074/jbc.M505806200P

[9] M. Giurg, K. Piekielska, M. Gȩbala, B. Ditkowski, M. Wolański, W. Peczyńska-Czoch, J. Młochowski, *Synth. Commun.* **2007**, *37*, 1779–1789. doi: 10.1080/00397910701316136

[10] R. R. Bhandare, B. E. Blass, D. J. Canney in *Medicinal Chemistry of Chemotherapeutic Agents* (Ed.: P. C. Acharya, M. Kurosu), Academic Press, London, Oxford, Boston, New York San Diego, **2023**, 115-133. doi: 10.1016/B978-0-323-90575-6.00013-2

[11] Z. Xie, L. Zhou, L. Guo, X. Yang, G. Qu, C. Wu, S. Zhang, *Org. Lett.* **2016**, *18*, 1402-1405. doi: 10.1021/acs.orglett.6b00332

[12] Y.-M. Hisiao, J.-L. Ko, C.-C. Lo*, J. Agric. Food Chem.* **2001**, *49*, 1669–1674. doi: 10.1021/jf0009192

[13] J. Jumper, R. Evans, A. Pritzel, T. Green, M. Figurnov, O. Ronneberger, K. Tunyasuvunakool, R. Bates, A. Žídek, A., Potapenko, A., Bridgland, C. Meyer, S. Kohl, A. J. Ballard, A. Cowie, B., Romera-Paredes, S. Nikolov, R. Jain, J. Adler, D. Hassabis, *Nature* **2021**, *596*, 583–589. doi: 10.1038/s41586-021-03819-2

[14] M. Varadi, S. Anyango, M. Deshpande, S. Nair, C. Natassia, G. Yordanova, D. Yuan, O. Stroe, G. Wood, A. Laydon, A. Zídek, T. Green, K. Tunyasuvunakool, S. Petersen, J. Jumper, E. Clancy, E., Green, A. Vora, M. Lutfi, S. Velankar, *Nucleic Acids Res.* **2022**, *50*, D439–D444. doi: 10.1093/nar/gkab1061

[15] Y. Matoba, T. Kumagai, A. Yamamoto, H. Yoshitsu, M. Sugiyama, *J. Biol. Chem.* **2006**, *281*, 8981–8990. doi: 10.1074/jbc.M509785200

[16] M. Sendovski, M. Kanteev, V. S. Ben-Yosef, N. Adir, A. Fishman, *J. Mol. Biol.* **2011**, *405*, 227–237. doi: 10.1016/j.jmb.2010.10.048

[17] Wingfield, *Curr. Protoc. Protein Sci.* **2017**, *88*, 6.14.1–6.14.3. doi: 10.1002/cpps.29
